# A systematic review on the effects of *Echinacea* supplementation on cytokine levels: Is there a role in COVID-19?

**DOI:** 10.1016/j.metop.2021.100115

**Published:** 2021-07-29

**Authors:** Monique Aucoin, Valentina Cardozo, Meagan D. McLaren, Anna Garber, Daniella Remy, Joy Baker, Adam Gratton, Mohammed Ali Kala, Sasha Monteiro, Cara Warder, Alessandra Perciballi, Kieran Cooley

**Affiliations:** a Canadian College of Naturopathic Medicine, Canada; b ph360.me/Shae, Australia; c University Technology, Sydney, Australia; d National Centre for Naturopathic Medicine at Southern Cross University, Australia; e Pacific College of Health and Science, United States

**Keywords:** Echinacea, Herbal medicine, Cytokine, Cytokine storm, Cytokine release syndrome, COVID-19, ARDS, acute respiratory distress syndrome, CCL, C–C motif ligand chemokine, COVID-19, coronavirus disease 2019, CSF, Colony-stimulating factor, GM-CSF, granulocyte-macrophage colony-stimulating factor, IFN, interferon, IL, interleukin, MCP, monocyte chemoattractant protein, MIP, macrophage inflammatory protein, SARS, Severe acute respiratory syndrome, SARS-CoV-2, severe acute respiratory syndrome coronavirus 2, TFN, tumor necrosis factor

## Abstract

COVID-19 is the respiratory illness caused by the novel coronavirus, SARS-CoV-2. Cytokine storm appears to be a factor in COVID-19 mortality. *Echinacea* species have been used historically for immune modulation. A previous rapid review suggested that *Echinacea* supplementation may decrease the levels of pro-inflammatory cytokines involved in cytokine storm. The objective of the present systematic review was to identify all research that has assessed changes in levels of cytokines relevant to cytokine storm in response to administration of *Echinacea* supplementation. The following databases were searched: Medline (Ovid), AMED (Ovid), CINAHL (EBSCO), EMBASE (Ovid). Title and abstract screening, full text screening, and data extraction were completed in duplicate using a piloted extraction template. Risk of bias assessment was completed. Qualitative analysis was used to assess for trends in cytokine level changes. The search identified 279 unique publications. After full text screening, 105 studies met criteria for inclusion including 13 human studies, 24 animal studies, and 71 *in vitro* or *ex vivo* studies. The data suggest that *Echinacea* supplementation may be associated with a decrease in the pro-inflammatory cytokines IL-6, IL-8, and TNF, as well as an increase in the anti-inflammatory cytokine IL-10. The risk of bias in the included studies was generally high. While there is currently no substantive research on the therapeutic effects of *Echinacea* in the management of either cytokine storm or COVID-19, the present evidence related to the herb's impact on cytokine levels suggests that further research may be warranted in the form of a clinical trial involving patients with COVID-19.

## Highlights

• Modulation of the immune system has been identified as a possible management strategy in severe COVID-19.
• A systematic review of all studies assessing changes in cytokine levels following *Echinacea* supplementation was undertaken.
• *Echinacea* supplementation may decrease the pro-inflammatory cytokines IL-6, IL-8, and TNF.
• *Echinacea* supplementation may increase the anti-inflammatory cytokine IL-10.
• Clinical trials assessing the effectiveness of *Echinacea* in the treatment of cytokine storm in COVID-19 may be warranted.

## 1 Introduction

In early January of 2020, severe acute respiratory syndrome coronavirus 2 (SARS-CoV-2) was identified as the agent responsible for coronavirus disease 2019 (COVID-19) [1]. As of June 2021, the global spread of this virus has led to a pandemic with approximately 176 million confirmed cases, including over 3.8 million deaths worldwide [2]. While the majority of COVID-19 patients experience mild to moderate flu-like symptoms (including fever, myalgia or fatigue, and dry cough), severe cases may lead to the development of complications such as acute respiratory distress syndrome (ARDS) and multiple-organ failure [3]. Current scientific literature suggests that “cytokine storm”’ is the main cause of ARDS and multiple organ failure in COVID-19 patients [4] through a pathologic process involving excessive inflammation and interference with coagulation leading to clot formation, organ tissue damage (notably in the lungs), multiple organ dysfunction syndrome, septic shock and ultimately death [1,5].

Cytokine storm, also known as cytokine release syndrome, is a phenomenon observed in response to a number of viral infections and is characterized by a rapid release of pro-inflammatory cytokines [6]. A recent literature review proposed a unified characterization of cytokine storm based on three criteria: “elevated cytokine levels, acute systemic inflammatory symptoms and secondary organ dysfunction beyond that which could be attributed to a normal response to a pathogen, if a pathogen is present'' [7]. Cytokines involved in cytokine storm include proinflammatory interleukin (IL)-6, IL-8, IL-1β, IL-12 and tumor necrosis factor (TNF), while other cytokines such as IL-10 inhibit the process through an anti-inflammatory effect [6]. When considering the role of cytokines in COVID-19 specifically, it has been observed that higher levels of IL-6, IL-8 and TNF, at the time of admission, were associated with significantly lower rates of survival after adjusting for demographics and comorbidities as confounding variables [8]. An association between higher IL-6 and IL-8 levels and increasing disease severity was also observed [8]. In another cohort of COVID-19 patients, highly impaired Interferon (IFN) type 1 response was consistent among severe and critically ill patients [9]. Decreased levels of INF-α and IFN-β were associated with ongoing elevation in blood viral load and an over-active response of pro-inflammatory modulators TNF and IL-6(9).

Given the central role of cytokine storm in the progression of severe COVID-19 cases, suppressing this immune response may be an opportunity to intervene. As such, several immunomodulatory treatments (including corticosteroids, Janus kinase (JAK) inhibitors, hydroxychloroquine, Tocilizumab and Colchicine) as well as antivirals like remdesivir and lopinavir/ritonavir have been proposed, but results have been mixed [[10], [11], [12], [13], [14]]. To date, only tocilizumab and dexamethasone have been shown to reduce mortality in severe COVID-19, while baricitinibe (a JAK inhibitor) is combination with remdesivir reduces recovery time [[15], [16], [17]]. Despite advances in treatment approach, severe COVID-19 remains challenging to treat and additional effective interventions are needed [[10], [11], [12], [13], [14]].

Herbal medicines, including species of *Echinacea*, have been used historically to modulate the immune system. The genus *Echinacea* has nine different species, with *Echinacea angustifolia, Echinacea pallida* and *Echinacea purpurea* commonly employed for medicinal purposes, notably as a treatment for various upper respiratory tract infections and inflammatory ailments [18]. Although the active constituents of the *Echinacea* genus are well known (e.g., polysaccharides, glycoproteins, caffeic acid derivative and alkamides), their exact mechanism of action is not well understood [[19], [20], [21]]. Nonetheless, this herbal therapy seems to be well tolerated with few adverse reactions reported [20].

Previous research indicates that the use of *Echinacea* may decrease the duration and severity of respiratory tract infections [18], making it a potential candidate to mitigate the symptoms of COVID-19. However, given its ability to stimulate the immune system, there are concerns that using this herb to treat COVID-19 could contribute to or exacerbate the potential for cytokine storm. Interestingly, a recent rapid literature review of clinical trials suggests that *Echinacea* may have the opposite effect, decreasing pro-inflammatory cytokines and increasing anti-inflammatory cytokines, which may provide a therapeutic benefit in the management of COVID-19(22). As such, the objective of the present systematic review is to identify all research that has assessed changes in levels of cytokines relevant to cytokine storm in response to administration of *Echinacea* supplementation.

## 2 Methods

### 2.1 Search strategy and databases

The following search terms were used: (Echinacea OR Echinacea angustifolia OR Echinacea purpurea OR coneflower) AND (Cytokine* OR cytokine storm OR cytokine release syndrome OR chemokine* OR interferon* OR interleukin* OR tumour necrosis factor* OR colony-stimulating factor*). The databases searched included Medline (Ovid), AMED (Ovid), CINAHL (EBSCO), EMBASE (Ovid). The search strategy was informed by an earlier rapid review [22] and conducted on July 14, 2020. An update of the search was conducted on April 12, 2021.

### 2.2 Study selection

Inclusion criteria: 1) administered *Echinacea*, 2) reported changes in levels of cytokine relevant to cytokine storm (at least one of the following: interferon, interleukin, chemokine, tumor necrosis factor, colony-stimulating factor) and 3) experimental or observational study design, including humans or animals, *in vitro/ex vivo* studies, and case reports. Exclusion criteria: 1) administration of *echinacea* in combination with other herbal, medical or nutritional supplements, 2) Reviews, systematic reviews, commentaries, and historical articles. Abstract and full text screening was completed independently in duplicate with any disagreement resolved by consensus.

### 2.3 Data extraction

Data extraction was completed using piloted extraction templates for human, animal, and cell culture studies. Complete study data was extracted by one reviewer. A second reviewer independently extracted outcome data and completed risk of bias assessment in duplicate; any disagreement was resolved by consensus. Predefined outcomes of interest included: changes in chemokines, interferon, interleukin, tumor necrosis factors, and colony stimulating factors, as well as the incidence of cytokine storm. The change in cytokine level reported in each study was extracted (i.e., increase, decrease or no change in cytokine production). The predefined study characteristics that were extracted from the human studies included: author, sponsorship, study design, study population, *Echinacea* species, *Echinacea* dose and duration, control or placebo, number of participants, inclusion/exclusion criteria, change in cytokine levels and incidence of cytokine storm. The characteristics extracted from the animal studies included: author, sponsorship, animal model, infection or method immune stimulation, *Echinacea* species, *Echinacea* dose, from and standardization, control or placebo, number of subjects, change in cytokine levels, and incidence of cytokine storm. The characteristics extracted from the cell culture studies included: author, sponsorship, cell or tissue culture, infection or method immune stimulation, *Echinacea* species, *Echinacea* dose, form and standardization, duration, control or placebo, change in cytokine levels, and incidence of cytokine storm.

### 2.4 Risk of bias assessment

Risk of bias assessment was completed using the following tools: Cochrane Risk of Bias 2.0 (randomized clinical trials) [23], ROBINS-I (non-randomized trials) [24], NIH Quality Assessment Tool (pre-post studies with no control group) [25], OHAT (animal studies) [26], and ToxRtool (*in vitro* studies) [27].

### 2.5 Data analysis

Studies were grouped based on methodology. The number of studies reporting increases, decreases or no change in each cytokine were counted and presented in figures to assess for trends visually. Statistical pooling was not feasible due to a qualitative assessment of heterogeneity made by the author team.

## 3 Results

**Fig. 1:**
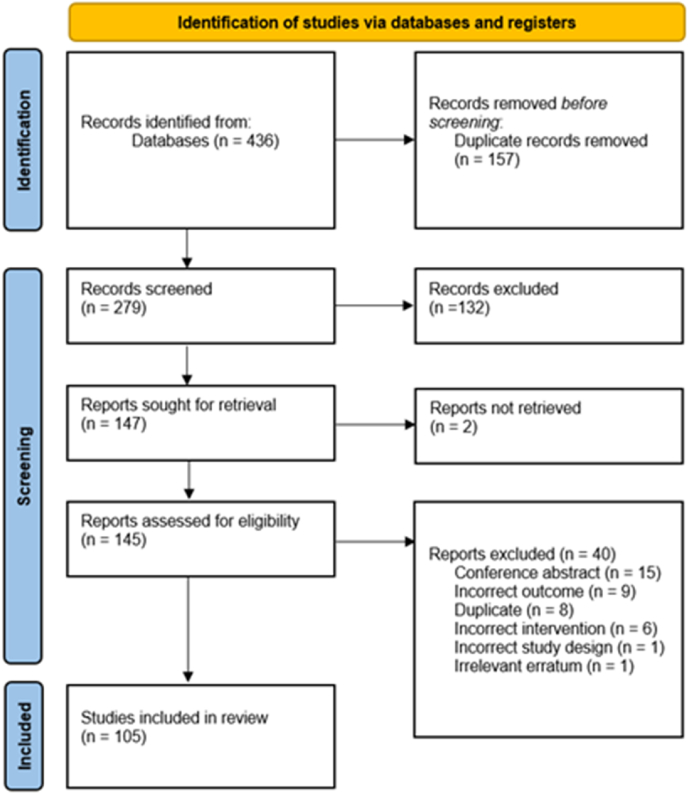
PRISMA flow diagram of included studies.

Of the 436 records identified, 105 studies met criteria for inclusion in the present systematic review (Fig. 1). Excluded studies are listed in Supplemental File 1. Of the 13 studies involving human participants, seven were randomized clinical trials [[28], [29], [30], [31], [32], [33], [34]], three were non-randomized trials [[35], [36], [37]] and three were pre/post uncontrolled trials [[38], [39], [40]]. Twenty-four studies reported outcomes related to animal experiments [[41], [42], [43], [44], [45], [46], [47], [48], [49], [50], [51], [52], [53], [54], [55], [56], [57], [58], [59], [60], [61], [62], [63]] and 69 studies reported outcomes related to *in vitro* or *ex vivo* studies [39,[64], [65], [66], [67], [68], [69], [70], [71], [72], [73], [74], [75], [76], [77], [78], [79], [80], [81], [82], [83], [84], [85], [86], [87], [88], [89], [90], [91], [92], [93], [94], [95], [96], [97], [98], [99], [100], [101], [102], [103], [104], [105], [106], [107], [108], [109], [110], [111], [112], [113], [114], [115], [116], [117], [118], [119], [120], [121], [122], [123], [124], [125], [126], [127], [128], [129], [130], [131]]. Table 1, Table 2, Table 3 present the characteristics and results of the human, animal and *in vitro/ex vivo* studies respectfully.

**Table 1:** Characteristics of the human studies included.

Author	Sponsorship	Design	Study Population	*Echinacea* Spp	Dose and Duration of Treatment	Control or Placebo	Number of participants in analysis	Inclusion/Exclusion criteria	Change in Cytokine Levels
Barrett 2010 [26]	National Center for Complementary and Alternative Medicine (NCCAM) of the National Institutes of Health (NIH).	Placebo controlled RCT (4 arms)	People 12–80 years of age, with new-onset common cold	*E. purpurea* and *E. angustifolia* root extracts	Four doses of 2 tablets within 24 h of enrollment (10.2 g of dried *echinacea* root). Followed by one tablet four times per day (5.1 g per day) for 4 days. 1 tablet = 675 mg of *E. purpurea* and 600 mg E*.angustifolia,* each standardized to 2.1 mg of alkamides. DURATION: 5 days	Visually matched placebo containing identical amounts of excipients (calcium acid phosphate, cellulose, silica, sodium starch glycollate, Hypromellose and magnesium stearate)	TOTAL: 713 INTERVENTION: 183 blinded & 181 unblinded PLACEBO: 173 unblinded & 176 blinded	INCLUSION: At least 1 of 4 common cold symptoms (nasal discharge, nasal obstruction, sneezing, or sore throat) and a score of 2 or higher on Jackson criteria. EXCLUSION: Use of antibiotics, antivirals, nasal steroids, decongestants, antihistamines, combination cold formulas, *echinacea*, zinc or vitamin C. History of allergic rhinitis and/or asthma. People with autoimmune/immune deficiency disease and pregnant women.	-Non statistically significant rise in mean nasal rinse IL-8 levels in both *echinacea* groups compared to placebo.
Isbaniah, 2011 [27]	Frutarom Switzerland Ltd.	Double-blind, placebo controlled RCT (3 arms)	COPD outpatients 40–81 years of age (mean age of 65.8)	*E. purpurea* from dried pressed juice of the aerial parts of the plant	500 mg of ciprofloxacin twice a day for 7 days and either tablets with 1) 500 mg E*. purpurea* or 2) 500 mg of *E. purpurea* with 10 mg zinc, 15 μg selenium and 50 mg ascorbic acid (EP+) once a day. DURATION: 14 days	Composition not stated	TOTAL: 108 INTERVENTION: 36 *Echinacea* only & 37 *Echinacea* with zinc, selenium and ascorbic acid PLACEBO: 35	INCLUSION: COPD outpatients 40+ years of age with an acute exacerbation episode (non-gradual increase in at least one major symptom: dyspnoea, sputum production and sputum purulence). EXCLUSION: History of asthma, severe immune system disorder, malignancy or haematologic disorder, obstructive pulmonary disease caused by other reasons or any other disease with known impact on COPD recovery. Increase of >/ = 12% of the pulmonary function after using a bronchodilator; severe clinical symptoms in addition to cor pulmonale and heart failure, utilization of extra respiratory muscles, and oxygen dependence (scale IV); requirement for treatment anti-inflammatory drugs; pregnancy or lactation; hypersensitivity to Echinacea or ciprofloxacin.	-No statistically significant change in IL, IL-10 or TNF-α serum concentration for *echinacea* only group compared to placebo. -IL1-β serum concentration significantly increased in both the *echinacea* only and placebo group (no difference between groups).
Turner, 2005 [28]	Supported by a grant (R01 AT001146) from the National Center for Complimentary and Alternative Medicine of the NIH	Double-blind, placebo controlled RCT (7 arms)	Healthy young adult (age 20.8 ± 3.3) volunteers exposed to rhinovirus experimentally	*E. angustifolia* root extract tincture extracted with either 1) supercritical CO2, 2) 60% ethanol or, 3) 20% ethanol	Dose: 1.5 mL of tincture containing 300 mg of echinacea extract three times a day. Two phases: 1) Prophylaxis - 7 days before viral challenge 2) Treatment- 5 days after viral challenge. Seven interventions: 1) One of three *echinacea* preparations during both prophylaxis and treatment 2) Placebo during prophylaxis and an *echinacea* preparation during treatment 3) Placebo during both prophylaxis and treatment. DURATION: 12 days	Mixture of alcoholic beverages, denatonium benzoate and tap water	TOTAL: 399 INTERVENTION: 48-52 per arm PLACEBO: 103	INCLUSION: Healthy young adults, susceptible to rhinovirus type 39 (based on antibody testing). EXCLUSION: Existing antibodies to test virus at screening or at day zero.	-Prophylaxis and/or treatment with three different *echinacea* preparations did not have a statistically significant effect on IL-8 in nasal lavage in response to infection when compared to placebo.
Kim, 2002 [29]	Celestial Seasonings inc, Larex inc, Lee Dexter and associates	Double-blind, placebo controlled RCT (6 intervention arms)	Healthy female volunteers 22–51 years of age (mean age 36.7)	*E. purpurea* whole herb extract (4% phenols), ultra-refined *E. purpurea* whole herb, *E. angustifolia* root, *E. purpurea* whole herb	Two capsules twice per day for a daily total of either: 1) 1500 mg of *E. purpurea* with 4% phenols (EP); 2) 780 mg of *E. purpurea* (4% phenols) and 680 mg of ultra-refined *E. purpurea* and *E. angustifolia* (urEPA); 3) 908 mg of *E. purpurea* (4% phenols), 464 mg of *E. purpurea*, and 36 mg of *E. angustifolia* (EPA); 4) 908 mg of *E. purpurea* (4% phenols), 464 mg of *E. purpurea*, 46 mg of *E. angustifolia* and 1500 mg of larch arabinogalactan; 5) 1500 g of larch arabinogalactan. DURATION: 28 days	Alfalfa and rice capsules matching in colour, size and taste.	TOTAL: 46 INTERVENTION: 8 per arm PLACEBO: 8	INCLUSION: Healthy adult females EXCLUSION: Major illness: cancer, diabetes, cardiovascular, autoimmune/immune diseases. Acute illness at enrollment/during study period including upper respiratory tract infections and sinusitis. Taking immune enhancing/altering supplements or medications.	-Statistically significant (p = 0.040) decrease in TNF-α serum concentration after 4-weeks of intervention in urEPA group. -No significant (p>0.05) decreases in TNF-α levels in groups taking EP, EPA or placebo.
Whitehead, 2007 [30]	Unlear	Double-blind, placebo controlled Randomized/matched trial	Healthy male volunteers, 24.9 ± 4.2 years of age, with 19.3% ± 6.5% body fat	*E. purpurea* extract from the aerial parts of the plant - Puritan's Pride®	Five 400 mg E*. purpurea* capsules four times per day for a total daily intake of 8 g per day. Daily multivitamin. DURATION: 28 days	Wheat flour and a multivitamin	TOTAL: 24 INTERVENTION: 12 PLACEBO: 12	INCLUSION: Healthy male students, age 18–30, deemed recreationally active (i.e., ≥30 min of physical activity 3 days/week). EXCLUSION: Taking medications, using dietary supplements or any form of tobacco, any sign/symptom of cardiovascular or metabolic diseases.	-IL-3 serum concentration increased significantly (p = 0.011) at day 14 (65% increase from baseline) and 21 (73% increase from baseline) in the *Echinacea* group compared to placebo group. -No significant changes in Granulocyte-macrophage CSF levels between *echinacea* and placebo groups.
Schwartz, 2002 [31]	Grants from Shaper & Bruemmer and two of the authors (C. Bode and J. C. Bode)	Double-blind, placebo controlled crossover RCT	Healthy male volunteers 28 ± 5.8 years of age, with a body mass index of 22.9 ± 2.1	*E. purpurea*, freshly expressed juice; identical to commercially available ESBERITOX™ mono	Unspecified amount of either juice or placebo two times per day for 14 days; 4-week washout period followed by 14 days of opposite intervention. DURATION: 14 days	Ethanol, water solution with artificial color and flavour mimicking *Echinacea* juice.	TOTAL: 40 INTERVENTION: 40 PLACEBO: 40	INCLUSION: Healthy men, 20–40 years old. EXCLUSION: Acute or chronic disease, known atopic diathesis, acute infection one month prior to the study, obesity (BMI >28), immunomodulating drugs (NSAIDs, smoking, excessive alcohol consumption).	-No statistically significant change in production of IL-1β from isolated blood monocytes. -TNF-α production of monocytes cultured with LPS did not differ between intervention and control groups (40 pg/mL detection limit).
Berg 1998	Unclear	Double-blind, placebo controlled RCT (3 intervention arms)	Healthy male triathletes 27.5 ± 5.3 years of age, with VO2 max>52mL/kg/min, undergoing regular training for triathlon sprint competition (mean 4.3 years)	E. purpurea pressed juice (Echniacin)	The following medications were taken daily, in three divided doses at meal times: 1) 8 mL of pressed echinacea juice (final concentration of 80 g in 22% ethanol) plus 12 flavoured placebo tablets or; 2)12 Magnesium tablets and 8 mL of flavoured 22% ethanol or; 3)12 flavoured tablets and 8 mL of flavoured 22% ethanol. DURATION: 28 days (prior to triathlon sprint competition)	Flavoured tablets and 120 drops (8 mL) flavoured 22% ethanol. Note: Magnesium group served as “a reference for supplementation with a nutrient required for optimal muscular function”. Each tablet contained 265 mg Mg (HPO4) 2*3H2O and 6 g Mg (hydrogen citrate) 2*3H2O	TOTAL: 40 INTERVENTION: 14 Echinacin 13 Magnesium PLACEBO: 13	INCLUSION: Male triathletes, 18–47 years old, free from any infection 2 weeks prior to the start of the study. EXCLUSION: Treatment with vitamin E (>200 mg/day) or other antioxidants, fish oil products, regular laxatives, tonics, corticosteroids, immunosuppressants, lipid lowering agents or anticoagulant drugs, and excessive alcohol use.	-All groups experienced a decrease in urine and serum sIL-2R and IL-6 1 h after the competition. After 24 h sIL-2R concentration remained low while IL-6 concentration returned to baseline. -Statistically significant (p < 0.05) decrease in serum IL-2R 1 h and 20 h after the competition in the Echinacin group compared to placebo. -Treatment with Echinacin resulted in a significantly more pronounced increase in urine IL-6 1 h after the competition, compared to placebo.
Obukhova, 2008 [32]	Unclear	Non-randomized, controlled, intervention study	Patients with clinical remission of chronic herpes infection, 17–52 years of age	Plant preparation of 60% *E. purpurea* and 40% *E. pallida* extracts (phytomicropheres).	Two *echinacea* capsules (unspecified amount) during day one (morning and evening). Then one capsule per day for four days. DURATION: 5 days	Patients with clinical remission of chronic herpes infection that did not receive *Echinacea* immune-corrective therapy.	TOTAL: 52 INTERVENTION: 38 CONTROL: 14	INCLUSION: Patients with clinical remission of chronic herpes infection (defined as absence of chronic inflammation at least one month before the trial). EXCLUSION: none included.	-IFN-γ, IL-1β and IL-6 plasma concentrations at baseline were above normal in the intervention and control groups (p < 0.05). -IFN-γ concentration in the intervention group increased significantly (p < 0.05) on day 7 post-treatment and continued to increase progressively on days 14 and 21 exceeding levels before and 7 days after therapy (p < 0.01 and p < 0.05, respectively). There were no statistically significant changes in IFN-γ plasma concentration in the control group. -IL-1β plasma concentration in the intervention group decreased significantly (p < 0.05) on day 7 post-treatment, then increased slightly (without exceeding pre-treatment levels) on days 14 and 21 post-treatment. There were no statistically significant changes in IL-1β plasma concentration in the control group. -IL-6 plasma concentration in patients of the treatment group decreased significantly (p < 0.05) on day 7 post-treatment, then increased back to baseline levels on day 14, and increased further on day 21 post treatment (p < 0.05). There were no statistically significant changes in IL-6 plasma concentration in the control group.
Roesler, 1991 [33]	Unclear	Non-randomized, controlled intervention study	Healthy volunteers 20–45 years of age	*E. purpurea* polysaccharides purified from large-scale cell cultures	Injection containing 5 mg of *E. purpurea* polysaccharides (2:1 xyloglucanes, arabinogalactane mixture). DURATION: Single dose	0.9% NaCL	TOTAL: 10 INTERVENTION: 5 CONTROL: 5	INCLUSION: negative history of allergies, autoimmune diseases, and severe diseases. EXCLUSION: none included.	-No statistically significant changes in IL1-β, IL-6, TNF-α or neopterin concentrations in serum and plasma between the *echinacea* and placebo groups.
Dapas, 2014 [34]	Italian Minister of Instruction, University and Research (MIUR), PRIN 2010, number 20109PLMH2.	Interrupted time series study (before-after study with control baseline).	Healthy adults (age 26–53) of both genders	*E. angustifolia* dry root extract (triple standardized extract syrup Polinacea®)	10 mL of syrup once a day (between meals) containing 100 mg of Polinacea (4.7 mg of echinacoside and 8.0 mg of high molecular weight polysaccharides). DURATION: 28 days	N/A	TOTAL: 10 INTERVENTION: 10 CONTROL: N/A	INCLUSION: Healthy individuals with normal liver function. No medicines taken one week before or during the study. Fasting at baseline. EXCLUSION: Smoking, dietary restrictions, allergy to Compositae or Grossulariacee plants.	-Statistically significant (p < 0.05) increase in IL-2 and decrease in IL-6 plasma concentrations post intervention. Non-statistically significant change in IL-8 (p = 0.08) and TNF-α (p = 0.58) plasma concentrations post intervention compared to baseline. -Statistically significant (p < 0.05) downregulation of TNF-α mRNA in circulating lymphocytes post intervention.
Guiotto, 2008 [35]	DALCO s.r.l. and the Region Friuli Venezia Giulia	Single blind crossover study (3 arms, no control group)	Healthy individuals of both genders	*E. purpurea* dry root extract	One lozenge (3 g) after overnight fasting containing glucose syrup, crystalline sugar and 100 mg of dry *E. purpurea* extract with either 0.7 mg, 0.21 mg or 0.9 mg of dodeca-2E,4E,8Z,10E/Z-tetraenoic isobutylamides. Doses were administered in increasing order with a 2-week washout period between them. DURATION: Single dose	N/A	TOTAL: 6 INTERVENTION: 6 CONTROL: N/A	INCLUSION: Healthy individuals. Abstinence from smoking, eating and drinking (only water allowed) starting 12 h before treatment and culminating 2 h post treatment. No medicine to be taken from one week before to the end of the study except for oral contraceptives. EXCLUSION: Dietary restrictions	-All three dose quantities led to a statistically significant (p < 0.05) decrease in IL-12p70, IL-8 and IL-6 plasma concentration 24 h post-intervention compared to baseline. The two larger doses also led to statistically significant decreases in IL 10 and TNF-α (p < 0.05), however the smallest dose did not (p = 0.059). 24 h after intervention the level of TNF-α was approximately 61% of the pre-treatment value, 68% for IL-6, 64% for IL-8, 73% for IL-10 and 76% for IL1-2p70.
Dall'Acqua, 2015 [36]	Farmaderbe, Pradamano (Udine) and Indena S.p.A. (Milan, Italy)	Single blind, before-after study without control group	Healthy adults (age 26–53) of both genders	*E. angustifolia* lipophilic root extract -Echinamid ®	One soft gel capsule (10 mg) after overnight fasting containing 1 mg of dodeca-2E,4E,8Z,10E/Z-tetraenoic isobutylamides, gelatin, glycerin, titanium dioxide, and iron oxide yellow. DURATION: Single dose	N/A	TOTAL: 10 INTERVENTION: 10 CONTROL: N/A	INCLUSION: Healthy individuals with normal liver function. Abstinence from smoking, eating and drinking (only water allowed) starting 12 h before treatment. No medicines to be taken during the study. EXCLUSION: Dietary restrictions, allergy or sensitivity to Compositae or Grossulariacee plants.	-Statistically significant (p < 0.05) decrease in IL-2, IL-6, IL-8, IL-10 and TNF-α plasma concentration 24 h post-intervention. -Statistically significant (p < 0.05) decrease in IL-2, IL-6, IL-8 and TNF-α mRNA/28S levels (measured via real time PCR). -Statistically significant (p < 0.05) increase in IL-10 mRNA levels.
Randolph, 2003 [37]	Unclear	Open label, before-after study without control group	Healthy adults (age 18–65) of both genders, weighing 55–79 kg.	*E. purpurea* (root and aerial parts) and *E. angustifolia* root extracts (NUTRILITE Triple Guard® *Echinacea* tablets)	Three tablets, three times daily (1518 mg/day) for two days, plus three tablets on day three (506 mg/day). 1 tablet = 252 mg of *E. purpurea* (aerial parts), 16 mg of *E. purpurea* (root), 12 mg of *E. angustifolia* (root) and 33 mg of Citrus Bioflavonoid (Citrus limon, C. paradisi, C. reticulate x, C. sinesis) DURATION: 2.5 days	N/A	TOTAL: 6 INTERVENTION: 6 CONTROL: N/A	INCLUSION: Adults (age 18–65), non-smoking, normally active, good health based on interview and physical examination. EXCLUSION: Smoking.	-Gene expression of IFN-α2 increased steadily through day 12 post-intervention in all subjects achieving statistical significance (p = 0.02) on day 12 (compared to baseline). -Small (non-statistically significant) down-regulation of IL-1β and IL-8 gene expression in some but not all subjects. -Small down-regulation in TNF-α gene expression in some but not all subjects. The magnitude of this downregulation achieved statistical significance (p = 0.04) on day 5 post-intervention but reverted toward baseline levels by day 12.

COPD: Chronic Obstructive Pulmonary Disease; EP; *Echinacea purpurea;* g: Grams; IFN: Interferon; IL: Interleukin; kg: Kilograms; mg: Milligrams; ml: Millilitres; NaCl: Sodium Chloride; NSAID: Nonsteroidal Anti-Inflammatory Drugs; RCT: Randomized controlled trial; TNF: Tumour Necrosis Factor; ug: Microgram.

**Table 2:** Characteristics of the animal studies included.

Author	Sponsorship	Animal Model	Infection or immune stimulation	Echinacea Spp or individual constituent	Dose, form, standardization	Control or Placebo formula used	Total Number of Subjects	Change cytokine levels
Abdelmonem, 2015 [38]	No financial support	Male Wistar rats, weighing 170 ± 20 g	Subcutaneous injection of isoprenaline (85 mg/kg) for 2 successive days (infarct-like myocardial lesion)	*E. purpurea*	*E. purpurea* (130 mg/kg) DURATION: 28 days	saline with no treatment; Isopropaline with no treatment	TOTAL: 84 INTERVENTION: 12 PLACEBO: 24	-no statistically significant change in IL-8 levels
Abdallah, 2015 [39]	Unspecified	Adult Sprague-Dawley rats, weighing 125–150 g	3 days of cyclophosphamide injection of 50 mg/kg/day	*E. purpurea* suspension cultures	Either 100 mg/kg or 200mg/kg oral dose of *E. purpurea* suspension cultures DURATION: 21 days	10 mg/kg of normal saline orally	TOTAL: 24 INTERVENTION: 6 per group (12 total) CONTROL: 6 saline only; 6 cyclophosphamide	-IL-1 statistically significant decrease in 200 mg/kg group -Statistically significant dose-dependent decrease in TNF-α
Abdel Rahman, 2018 [40]	No financial support	Nile Tilapia, 65–91 g	None	Dry extract of *E.purpurea*	500 mg E*.purpurea* /kg twice daily DURATION: 28 days	Basal diet	TOTAL 120 INTERVENTION: 30 in *E.purpurea* group (remaining animals received other herbs) PLACEBO: 10	-No difference in IL-1β expression -Statistically significant decrease in TNF-α expression in head kidney but not intestine
Cundell, 2003 [41]	Philadelphia University	Male Sprague- Dawley rats, 12 months of age	None	*E. purpurea* extract from aerial parts	1.05 g *E. purpurea*, 10.5 mg cichoric acid combined with gelatin and water for a total daily intake of 50 mg/kg of *Echinacea* and 0.5 mg/kg cichoric acid). DURATION: 8 weeks	Peanut butter	TOTAL: 16 INTERVENTION: 8 PLACEBO: 8	-increase in circulating IL-2 levels during weeks 4–5
Dogan, 2014 [42]	No financial support	Male Wistar-Albino rats, weighing 200–250 g	Acute colitis induced by 4% acetic acid	100 mg E*. angustifolia* & 400 mg E*. purpurea*	50 mg/kg of *Echinacea* per day using a catheter to rats DURATION: 14 days	Either acetic acid and saline or no acetic acid and no treatment	TOTAL: 20 INTERVENTION: 5 per group (colitis; no colitis) PLACEBO: 5 per group (colitis; no colitis)	-significantly decreased IL-1β (p < 0.007) -significantly decreased TNF-α p < 0.001)
Fusco, 2010 [43]	Weill Cornell Medical College Clinical and Translational Science Center (NIH), Stony-Wold Herbert Fund, National Center for Complementary & Alternative Medicine	Female C57BL6 mice, 6–8 weeks of age, 15–20 g	Influenza A/WSN/33 (H1N1) strain	*E. purpurea* Ethanol extracts freeze-dried to powder form	10 mg (100 μl of stock solution) administered to mice daily by gavage DURATION: 5 days	PBS	TOTAL: 59 INTERVENTION: 15 PLACEBO: 34	-Statistically significantly lower IFN-γ in serum (p-0.01), not lung (p = 0.3) -Statistically significantly lower IL-10 in serum and lung, decreased IL-5 and IL-12 on day 3, no statistically significant diff in IL-1β, IL-2, IL-4 -TNF-α No statistically significant diff
Ghaemi, 2009 [44]	Unspecified	Female BALB/c mice, 4–5 weeks of age, with an average weight of 20 g.	Live KOS strain of HSV-1 on Day 0 and 21	*E. purpurea* extract, concentration of 20 mg/mL	100 g of *E. purpurea* extract *E. purpurea* extract *E. purpurea* extract *E. purpurea* extract DURATION: 28 days	PBS inoculation or HSV-1 only	TOTAL: 30 INTERVENTION: 10 PLACEBO: 20	-increased IFN-γ (p-value not reported)
Goel, 2002 [45]	Unspecified	Male Sprague Dawley rats weighing 425–475 g	LPS	Cichoric acid, polysaccharide and alkylamide fractions	Group B: 40mcg/kg/day of Cichoric acid, 1000mcg/kg/day polysaccharide and 4mcg/kg/day alkylamide as oral gavage twice a day. Groups C, D & E got 3, 20 & 50 times this amount. DURATION: 4 days	50% ethanol	TOTAL: 30 INTERVENTION: 24 PLACEBO: 6	-Statistically significant increase IFN-γ (p < 0.05) at highest dose (50 times the extract level) -No effect on IL2- release -Statistically significant increase in TNF-α production at higher doses (50 times the extract level) (p < 0.05).
Goel, 2002 [46]	Unspecified	Male Sprague–Dawley rats, weighing 225–275 g	LPS	Cichoric acid, polysaccharide and alkylamide fractions	Oral gavage twice a day for 4 days of either: 1) cichoric acid (5–120mg/kg/day); 2) polysaccharides (125–3000mg/kg/day); or 3) alkylamides (0.5–12mg/kg/day) DURATION: 4 days	50% ethanol	TOTAL: 60 INTERVENTION: 54 PLACEBO: 6	-No Statistically significant effect on the release of IFN-γ by the rat splenocytes was observed -No statistically significant effect from any extract on IL-2 -Statistically significant increase in TNF-α production after exposure to polysaccharide and alkylamide (p < 0.05) but not cichoric acid
Hayashi, 2001 [47]	No financial support. The *E. purpurea* preparation was donated by API Companey, Gifu, Japan.	Female AKR/J mice, 3–4 weeks of age	Thymic injection of recombinant Leukemia Viruses from thymuses inducing leukemia	70% ethanol extract from partially purified powder from the leaves of *E. purpurea*	Oral 0.25 mg/ml EP suspended in PBS 3 times per week for 8 weeks amounting to 75mg/kg/week. DURATION: 24 weeks	Oral PBS	TOTAL: 20 INTERVENTION: 10 PLACEBO: 10	-Production of IFN-γ in the peritoneal exudate increased. No p-value reported -Modest production of IL-12, no p-value reported -Modest production of TNF-α, no p-value reported
Jiang, 2014 [48]	Key Nature Science Foundation for Colleges and Universities of Anhui Province of China and Anhui Agricultural University	Male Sprague Dawley rats, 160–200 g	Collagen-induced arthritis	Cichoric acid extract	Either 8, 16, or 32 mg/kg/day orally DURATION: 28 days	Tripterygium glycosides tablet (10 mg/kg/day)	TOTAL: 60 INTERVENTION: 10 per group (30 total) PLACEBO: 30	-Statistically significant reduction in IL-1β in serum (p < 0.01) -Statistically significant reduction of TNF-α in serum for all doses, only 32 mg/kg reduced in synovium
Liu, 2012 [49]	National Science Foundation of China, China National “863″ program	Kunming mice (weighing 14–16 g) and dogs (weighing 5–8 kg, 3–4 months of age)	Rabies vaccine	*Echinacea* polysaccharide containing 80% glucose	Injection of polysaccharides added to vaccine at 2 mg/mL for mice and 10mg/mL for dogs DURATION: 14 days for mice, 6 months for dogs	vaccine without polysaccharides	TOTAL: 250 mice and 30 dogs INTERVENTION: 50 mice per group (150 total), 6 dogs per group (24 total) PLACEBO: 50 control mice, 6 control dogs	-Statistically significant increase in IFN-γ response. Statistically significant increase in IFN-α (p < 0.05). -Enhanced release of cytokines within 1 day after inoculation. Includes IL-1β, IL-5 and IL-6. Statistically significantly higher than those in the control group (p < 0.05).
Liu, 2017 [50]	National Key Research and Development Program of China, National Natural Science Foundation of China, Scientific Startup Funds for Doctors of Northwest Agriculture and Forestry University	C57BL/6J mice, 3 months of age	0.25mg/kg/day LPS injection	Chicoric acid	0.05% Chicoric acid in drinking water DURATION: 54 days	Healthy control or LPS-induced	TOTAL: 30 INTERVENTION: 10 PLACEBO: 10 per group (20 total)	-serum IL-1β inhibited, and suppressed upregulation of L-6, IL-1β mRNA, but promoted IL-10 mRNA expression -serum TNF-α inhibited and suppressed upregulation of its mRNA expression
Li, 2020 [51]	Key Research and Discovery Program of Shandong Province, National Natural Science Foundation of China, High-Level Talent Research Foundation of Qingdao, Agricultural University, China, Chinese Herbal Medicine Industry Innovation Team of Shandong Province, Agricultural Technology System.	Male BALB/C mice (6–8 weeks old)	LPS induced Immune stimulation	*E. purpurea* aerial parts	50 mg per g IP injection of polysaccharides (30 min before LPS injection). DURATION: 8 h	Saline	TOTAL: 18 INTERVENTION: 6 CONTROL: 6 LPS only, 6 saline only	-Statistically significant decreased secretion of IL-6 and TNF-α (p < 0.05) -Statistically significant increased secretion of IL-10 (p < 0.05)
Park, 2018 [52]	Frutarom, Switzerland; Novarex, Republic of Korea; and Program for Industrial Needs - Matched Education (PRIME), Ewha Womans University funded by the Ministry of Education of Korea	Male BALB/c mice, 6 weeks of age, weighing 18–20 g	Restraint-induced immunosuppression	Cold pressed *E. purpurea* juice with extract ratio of 40–50:1	*E. purpurea* at doses of 10, 30, and 100 mg/kg of body weight DURATION: 2 weeks	0.9% saline	TOTAL: 70 INTERVENTION: 14 per group (42 total) CONTROL: 0.9% saline	-Statistically significant reduction of IL-6, IL-10, and IL-17 and downregulated their mRNA expression (p < 0.05, p < 0.01, and p < 0.01, respectively)
Sgorlon, 2016 [53]	Nutrigene S.r.l. from the University of Udine, Italy	Medium to large sized dogs >2 years of age	None	*E. angustifolia*	2% extract at 5 mg/kg daily DURATION: 60 days	Food without nutraceuticals	TOTAL: 74 INTERVENTION: 14 in *Echinacea* group CONTROL: 21	-Statistically significant up regulation of CXCL8 expression (p < 0.01) -Statistically significant down regulation of TNF-α (p < 0.05)
Shi, 2020 [54]	National Natural Science Foundation of China, Third Batch of Giant Project of Hebei Province, Top Talent Project for Youths of Hebei Province, Doctoral Startup Foundation of Hebei Normal University of Science and Technology, High School Hundred Excellent Innovation Talent Program of Hebei Province, Natural Science Foundation of Hebei Province, Project of Department of Science and Technology of Hebei Province	Male c57BL/6 mice (8-week-old, 20 g)	LPS induced Immune stimulation	*E. purpurea* (90.26% purity)	5 or 10 mg per kg, with or without LPS DURATION: 1 day	No treatment	TOTAL: 30 INTERVENTION: 18 CONTROL: 6 no treatment, 6 LPS only	-Statistically significant downregulation of IL-1β, IL-6, and TNF-α
Sutovska, 2015 [55]	BioMed, Slovak GrantAgency VEGA, APVV agency, MZ	Adult male Trik strain guinea pigs, weighing 200–350 g	Ovalbumin exposure causing allergic airway inflammation	*E. purpurea* extract	Oral *Echinacea* complex (50 mg/kg) DURATION: 14 days	Either 1) saline, 2) salbutamol, 3) budesonide, or 4) healthy controls	TOTAL: 50 INTERVENTION: 10 PLACEBO: 40	-Statistically significant decrease in IL-4, IL-5, IL-13 in both bronchoalveolar lavage fluid and serum -Statistically significant decrease in TNF-α in both bronchoalveolar lavage fluid and serum (p < 0.001)
Turkistani, 2019 [56]	Unspecified	Male rats Sprague Dawley (180–210 g)	CISP induced renal toxicity	*E. purpurea* root liquid extract	Oral *E. purpurea* with 500 mg/kg/day for four weeks, on the day 21st received a single IP injection of CISP DURATION: 4 weeks	No treatment or CISP only	TOTAL: 40 INTERVENTION: 10 EP only, 10 EP + CISP CONTROL: 20	-Statistically significant increase in IL-10 (p < 0.001) -Statistically significant decrease in TNF-α (p < 0.001)
Uluisik, 2012 [57]	The Scientific Research Projects Coordination Unit of Selcuk University	Male Fisher rats, 6 weeks of age	None	*E. purpurea* root powder	Pellets with 0.75 g/kg of *E. purpurea* root powder DURATION: 40 days	Standard rat pellets	TOTAL: 48 INTERVENTION: 16 *echinacea* echinacea CONTROL: 16 control	-No Statistically significant diff in IL-10 mRNA expression -TNF-α mRNA expression Statistically significant higher than control on 20th day but not 40th day
Yamada, 2011 [58]	Unspecified	Male Sprague Dawley rats, 4 weeks of age	ConA mitogen	Ethanol extracts of *E. purpurea*	10 g of *Echinacea*, per kg of rat feed DURATION: 4 weeks 4 weeks	Experimental diet without herb	TOTAL: 40 INTERVENTION: 30 PLACEBO: 10	-Statistically significant increase in IFN- γ secretion -IL-2: Statistically significantly increased production; IL-4 Statistically significantly increased production (with ConA immune stimulation only); IL-6 Statistically significantly decreased (with ConA immune stimulation only) -Significant decrease in TNF-α production
Yu, 2013 [59]	Key National Sciences Foundation of Colleges and Universities, Anhui Province	Male Kunming mice weighing 18–22 g, male Wistar rats weighing 180–220 g	Xylene induced ear edema on mice, or egg albumin induced paw edema on rats, or cotton-induced granuloma on rats	*E. purpurea* essential oil	2.5 g, 5 g or 10 g of crude drug/kg/kgg/kg DURATION: 7 days	33 mg aspirin or saline	TOTAL: 120 rats (60 per type of infection) and 60 mice INTERVENTION: 10 per dosage group (90 total) CONTROL: 10 normal control, 10 model control, 10 aspirin (90 total)	-IL-6 levels were Statistically significantly reduced in the low dose group (p < 0.05). In the high dose group, IL-2 levels were increased (p < 0.05). -TNF-α statistically significant reduced at high dose (p < 0.05).
Zhai, 2007 [60]	National Institute of Environmental Health Sciences, Office of Dietary Supplements, National Institutes of Health	Male BALB/c mice, 8 weeks of age	Mitogen stimulation	Ethanol extracts from the dried roots of *E. angustifolia*, *E. pallida*, and *E. purpurea*	Oral gavage of 130 mg/kg of body weight once daily DURATION: 7 days	5% ethanol gavage	TOTAL: Not reported INTERVENTION: Not reported CONTROL: Not reported	-Statistically significantly increased IFN-γ production (p < 0.035) -All 3 preparations inhibited the release of IL-1β (p = 0.007). Only *E. angustifolia* and *E. pallida*-treated mice demonstrated statistically significantly higher production of IL-4 (p = 0.046) and increased IL-10 production (p = 0.057) -no effect on IL-6 by any of the preparation -Statistically significantly increased IL-2 (p < 0.035) -no effect on IL-12 production -Statistically significant inhibition of TNF-α production from splenocytes from all 3 preparations. (p = 0.004)
Zhang, 2020 [61]	National Natural Science Foundation of China, Third Batch of Giant Project of Hebei Province, Top Talent Project for Youths of Hebei Province, Doctoral Startup Foundation of Hebei Normal University of Science and Technology, High School Hundred Excellent Innovation Talent Program of Hebei Province, Central Committee Guides Local Science and Technology Development Project, Natural Science Foundation of Hebei Province	Male C57BL/6 mice 8 weeks old, 18–22 g	LPS induced immune stimulation	*E. purpurea*	5 or 10 mg per kg DURATION: 24 h	Saline	TOTAL: 30 INTERVENTION: 6 LPS + EP 5 mg/kg, 6 LPS + EP 10 mg/kg CONTROL: 6 LPS only, 6 EP 10 mg/kg only, 6 saline only	-Statistically significant dose-dependent decrease in IL-1β, IL-6, and TNF-α (all p < 0.01)

CISP: Cisplatin; ConA: Concanavalin A; CXCL: Chemokine Ligand; EP: *Echinacea Purpurea;* g: Grams; HSV-1: Herpes Simplex Virus-1; IFN; Interferon; IL: Interleukin; IP: Intraperitoneal; kg: Kilogram; LPS: Lipopolysaccharide; mcg: Microgram; mg: Milligram; mL: Millilitres; PBS: Phosphate-buffered Saline; TNF-α; Tumour Necrosis Factor alpha; μl: Microlitres.

**Table 3:** Characteristics of the *in vitro* and *ex vivo* studies included.

Author	Sponsorship source/association	Cells or tissue culture	Infection or immune stimulation	Echinacea Spp or individual constituent	Dose, form, standardization, Duration of treatment	Control or Placebo formula used	Change in cytokines	Risk of Bias^a^
Altamirano-Dimas, 2007 [62]	Not stated	The tracheo-bronchial line BEAS-2B and the rhinovirus-sensitive H-1 derivative of HeLa cells	Human rhinovirus type 14	*E. purpurea*	Two extracts: E1: an expressed juice extract of the aerial parts of E. purpurea E2: a 55% EtOH tincture, prepared with *E. purpurea* roots (1:9 w/v) Dose: 100 μg/mL of E1 or 50 μg/mL of E2 DURATION: 18 h	Negative control: no treatment on uninfected cells Positive control: no treatment on virally infected cells	Increased genetic expression: IL-8, IL-1RN, CSF2 Decreased genetic expression: TNF-α	3
Altamirano-Dimas, 2009 [63]	Not stated	The tracheo-bronchial line BEAS-2B and the rhinovirus-sensitive H-1 derivative of HeLa cells	Rhinovirus type 14	*E. purpurea*	Two extracts: E1: an aqueous expressed juice extract of the aerial parts of *E. purpurea* E2: a 50% EtOH tincture, prepared with *E. purpurea* roots (1:9 w/v) Dose: 100 μg/mL of E1 or 50 μg/mL of E2 DURATION: 18 h	Negative control: no treatment on uninfected cells Positive control: no treatment on virally infected cells	Increased gene transcription: IL-1β, IL-13, IL-6, CXCL5, CXCL1, CXCL2, CXCL12, CXCL13, CXCL14, CXCL5, CXCL4, CXCL8, CCL4, CCL2, GM-CSF Decreased gene transcription: IL-1α, IL-4, IL-10, IL-12, IL-16, CXCL9, CXCL1, CXCL2, CXCL11, CXCL5, CXCL4, CXCL8, CXCL17, CXCL12, CXCL18, CXCL4, CCL5, CCL7, CCL8, CCL2, CCL4, TNF-α	3
Benson, 2010 [64]	This project was supported by grants from NSF-EPSCoR (EPS-0091995) and NCRR (P20RR17670). NCRR is a component of the NIH.	Bone marrow-derived dendritic cells from C57BI/6 mice	OVA-FITC (10 μg/mL)	*E. purpurea*	2 extracts were prepared using the leaf and root with 75% EtOH as the solvent. Root extract doses: 150 μg/mL and 450 μg/mL Leaf extract doses: 50 μg/mL and 150 μg/mL DURATION: 48 h	Negative control: 0.5% EtOH	Increased: IL-6 and TNF-α	3
Brovelli, 2005 [65]	Not stated	TPH-1 cells	LPS (500 ng/mL)	*E. purpurea*	*E. purpurea* was harvested at various stages of plant development, aerial parts were dried, and extracts were created from dried parts and the solvent 50% DMSO/30% EtOH/20% water. Dose: 100 μg/mL DURATION: 6 h	Negative control: no treatment Positive control: LPS (500 ng/mL)	Increased production: IFN-γ, IL-1α, IL-1β, IL-8, MIP-α and TNF-α Decreased production: IL-10	3
Burger, 1997 [66]	Not stated	Human peripheral blood macrophages (isolated from a 50-year-old female)	LPS (5 μg/mL)	*E. purpurea*	Two 20% EtOH commercial preparations: *echinacea* fresh pressed juice and *echinacea* dried juice Fresh pressed juice doses: 10, 3.0, 1.2, 0.2, and 0.05 μg/mL Dried juice doses: 10, 1.0, 0.I, 0.03, and 0.01 μg/mL DURATION: 18, 36, or 72 h	Negative control: no treatment Positive control: LPS (5 μg/mL)	Increased secretion: IL-1, IL-6, IL-10 and TNF-α	1
Cadiz, 2019 [67]	University of Minnesota Undergraduate Research Opportunity Program and the Office of the Vice President for Research of the University of Minnesota (UMM Faculty Enhancement Research Fund).	Splenocytes from C57BL/6J wild-type mice	ConA (5 μg/mL for full dose, 5×10^-3 μg/mL for suboptimal dose)	*E. purpurea*	*E. purpurea* root extract Doses: 0, 0.1, 1, and 10 mg/mL DURATION: 24 or 48 h	Negative control: No treatment on ConA-stimulated cells	Increased levels: TNF-α No change in levels: IFN-γ and IL-2	3
Canlas, 2010 [68]	Not funded	BEAS-2B and Human skin fibroblasts	Leishmania donovani Rhinovirus type 1A	*E. purpurea*	Standardized commercial extract: Echinaforce, A. Vogel/Bioforce Dose used not specified DURATION: 48 h	Positive control: LPS (10 μg/mL)	Decreased concentration: IL-6 and IL-8	1
Cech, 2006 [69]	NIH NCCAM (Grant No. K01 AT00065–01, T32-AT00815, and R15 AT001466-01) and Research Corporation (grant No. CC5972).	Leukemic human T-lymphocytic cells (Jurkat E6.1 clone)	PHA and PMA	*E. purpurea* and dodeca-2E,4E,8Z,10Z-tetraenoic acid isobutyl- amide	EtOH extract was prepared from *E. purpurea* roots. Dodeca-2E,4E,8Z,10Z-tetraenoic acid isobutyl- amide was obtained from Chromadex; Santa Ana, CA, USA. Two *E. purpurea* doses containing 4 or 0.9 μg/mL of dodeca-2E,4E,8Z,10Z-tetraenoic acid isobutyl- amide Two dodeca-2E,4E,8Z,10Z-tetraenoic acid isobutyl- amide doses: 1.8 or 0.19 μg/mL DURATION: 2 h	Controls included cells with media alone, stimuli alone, and microsome reagents both with and without NADPH.	Decreased concentration: IL-2	1
Cech, 2010 [70]	UNC Research Competitiveness Fund	Murine RAW 264.7 macrophage-like cells	Influenza strain A/PR8/34	*E. purpurea* and alkylamides 4 (undeca-2E,4Z-diene-8,10-diynoic acid isobutylamide), 11a/b (dodeca-2E,4E,8Z,10E/Z-tetraenoic acid isobutylamide), 15 (dodeca-2E,4E-dienoic acid isobutylamide), and 16 (undeca-2E-ene-8,10-diynoic acid isobutylamide)	17 extracts: *E. purpurea* roots were harvested from 17 cultivation sites across North Carolina, pulverized into a fine powder, macerated for seven days in 75% EtOH at a ratio of 1:5 (g plant material: mL solvent), pressed, and filtered. Dose of extract #7 used in general cytokine and chemokine experiments: a dilution of 85% EtOH (precipitated) extract was used to produce a final concentration of 22 μm dodecatetraenoic acid isobutylamide (11a/b). Dose of extracts used in TNF-α experiments: 6.7 μL of 75% EtOH extracts and 5.8 μL of 85% EtOH (precipitated) extracts Doses of alkylamides: 0, 6.25, 12.5, 25, and 50 μg/mL DURATION: 24 h	Negative control: no treatment on uninfected cells Positive control: no treatment on infected cells	Increased production: IL-12p70 Decreased production: 1L-13, CXCL5, CCL2, CCL3, CCL5, CCL9, TNF-α No change in production: IL-4 and CCL1	1
Chicca, 2009 [71]	Not stated	Human peripheral blood mononuclear cells	LPS (350 ng/mL)	*E. purpurea*	Three extracts obtained from A. Vogel Bioforce AG, Switzerland: herba, root, and combo herba + root in a ratio of 95:5 Doses: herba extract (9.5 μg/mL), radix extract (0.5 μg/mL), and comb herba + radix extract (10 μg/mL) DURATION: 18 h	Positive control: LPS alone	Increased levels: IL-10 and TNF-α	1
Chiu, 2010 [72]	Genomics and Proteomics Program, Academia Sinica (AS94F002); National Science Council (96-2320-B-001-008), Taiwan, Republic of China; China Medical University and Hospital (DMR-97-143); Taiwan Department of Health Clinical Trial; Research Center of Excellence (DOH99-TD-B-111- 004)	Human myelogenic leukemia cell line THP-1	LPS (1 μg/mL)	*E. purpurea*	Extract: Butanol partitioned fraction of the stem + leaf of the *E. purpurea* Dose: 100 μg/mL DURATION: 0.5, 4 or 12 h	Positive control: LPS alone	Increased genetic expression: IL-5, IL-IR2, CXCR4, CCR1 and CCR8 Decreased genetic expression: IL-1β, IL-4, IL-13, IL, TNF-α, CCR2,CCR3,CCR4, CCL2, CCL4, CCL8, CCL22 and CXCR4	3
Classen, 2006 [73]	Not stated	Alveolar mouse macrophages	LPS (30 μg/mL)	*E. purpurea*	Seeds from *E.purpurea* were treated with absolute EtOH and a 1:10 dilution of deomestos Dose not stated. DURATION: 24 h	Negative control: no treatment Positive control: LPS (10 μg/mL)	Increased production: IL-6	3
Codorean, 2010 [74]	National Institute of Pathology, Bucharest	Human peripheral whole blood	5 mg/mL PHA, 2,5 mg/mL ConA, 50 ng/mL LPS	*E. purpurea*	15 mg/mL standardized extract DURATION: 48 h	Ech was the positive control. Exposure to a cytotoxic compound used as a negative control	Increased production: IL-2 No change production: IL-1β	3
Dong, 2006 [75]	Grant from the National Science Council of Taiwan (NSC91-3112-P-001-035-Y).	Jurkat leukemic T-cells	Anti-CD3 plus anti-CD28 (CD28-dependent stimulation) and PMA plus ionomycin (CD28^−^ independent stimulation)	*E. purpurea* and cynarin	Crude water extract of *E. purpurea*. Cynarin was extract from the crude extract using high performance liquid chromatography Dose for both: 100 μg/mL DURATION: 24 h	Negative control: PMA and ionomycin or anti-CD3 and anti-CD28 Positive control: FK506 (1 μg/mL)	Decreased production: IL-2	1
Fan, 2021 [76]	Grants the Jilin Scientific and Technological Development Program for the financial support and the National Natural Science Foundation of China	Mouse macrophages	LPS (0.1 μg/mL)	*E. pallida* and *E. purpurea*	Advantagoues roots of *E.pallida* (11.4 g) and *E.purpurea* (8.6 g) were cut into approx 1 cm length DURATION: 24 h	Negative control: No treatment	Decreased production: IL-6 and IL-1β	1
Farinacci, 2009 [77]	PRIN2005, Research Unit Bruno Stefanon	Ovine neutrophils	PMA	*E. angustifolia*	Standardized hydroethanolic extract called Polinacea that was prepared by the authors using a patent Extracts doses used: 0, 20, and 60 μg/mL DURATION: 1 or 22 h	Negative control: no treatment	Increased gene expression: IL-8	1
Fonseca, 2012 [78]	Integrative Medicine Service, Memorial Sloan-Kettering Cancer Centre	Jurkat T-cells	PMA plus ionomycin and Ionomycin	*E. purpurea*	Various concentrations Extract doses used: 0,10,25, 100 and 250 μg/mL DURATION: 40 min and 24 h	Untreated cells	Increased production: IFN-γ and IL-2	1
Fonseca, 2014 [79]	NIH NCCAM and ODS:1-P50-AT02779 Botanical Research Center for Botanical Immunomodulators, NIH NCI Cancer Education and Career Development R25 CA105012: Nutrition and Cancer Prevention and the Children's Cancer and Blood Foundation	Human Jurkat T-cells (cell line e6-1)	PMA and/or ionomycin	*E. purpurea*	Extract: fresh aerial parts were extracted with water, ethanolic precipitation, and size-exclusion chromatography Extract doses used: 0, 10, 25, 100 and 250 μg/mL DURATION: 40 min and 24 h	Negative control: FK506 (1 μg/mL in DMSO)	Increased concentration: IFN-γ and IL-2	1
Fu, 2017 [80]	National Natural Science Foundation of China (No. 31472128).	Murine bone marrow-derived macrophages	LPS (10 ng/mL)	E. purpurea	Extract obtained from Shandong Qilu Animal Health Co., Ltd. Chemical composition of extract: cichoric acid (3.045%), caftaric acid (1.575%), chlorogenic acid(0.065%), Nndeca-2Z,4E-diene-8,10-diynoic acid isobutylamide (1.635%). Dose: 100 μg/mL DURATION: 12 or 24 h	Negative control: no treatment Positive control: IFN-γ (10 ng/mL) + LPS (10 ng/mL) or IL-4 (20 ng/mL)	Increased secretion: IFN-γ, IL-1α, IL-6 and TNF-α	1
Groom, 2007 [81]	Charles River Laboratories Preclinical Services Montreal Inc.	Macrophages (cell line J774A.1) and NK cells (IL-2-dependent NK-92 cell line)	LPS (3 μg/mL)	*E. purpurea*	Standardized extract of *echinacea* (4% total phenolics) obtained from Stryka Botanics Co., Inc., Hillsborough, NJ. Dose: 0.128, 0.385, and 1.28 mg/mL DURATION: exact duration not stated	Positive control: LPS (3 μg/mL) for macrophages and IL-12 (3 U/ml) for NK cells	Increased synthesis: IFN-γ No change in synthesis: IL-12	3
Guidetti, 2016 [82]	Not stated	Human peripheral blood mononuclear cells [from 10 healthy volunteers] and canine peripheral blood mononuclear cells [from 10 healthy dogs]	PMA and ionomycin	*E. purpurea*	*E. purpurea* dried extract, polyphenols content min 4%, dissolved in EtOH and water. Dose not specified DURATION: 10–12 h	Positive control: stimulation with no treatment	Decreased production: IFN-γ No change in production: IL-4	3
Gulledge, 2018 [83]	Grants from the National Center for Complementary and Integrative Health, a component of the National Institutes of Health (1R15AT007259), the National Institutes of Health (R01 HD072968 to AJM), the Research and Innovation Seed Fund at North Carolina State University, the Departments of Biological Sciences and Chemistry at North Carolina State University, and the Comparative Medicine Institute at North Carolina State University.	RBL-2H3 cells, a basophilic leukemia cell line	Calcium ionophore A23187	*E. purpurea* root extract and alkylamide dodeca-2E,4E-dienoic acid isobutylamide (A15)	Alkylamide dodeca-2E,4E-dienoic acid isobutylamide was synthesized and used in doses of 25, 50 and 100 μM DURATION: 8 h	Stimulation with A23187 without A15	Decreased production: TNF-α	1
Hou, 2010 [84]	Institutional grant of Academia Sinica and national research program for genomic medicine (NSC 97-3112-B-001-020) of National Science Council of Taiwan, R.O.C.	Murine macrophage RAW 264.7 cells	LPS (1.0 μg/mL)	*E. purpurea*, dodeca-2E,4E,8Z,10Z(E)-tetraenoic acid isobutylamide, and cichoric acid	A series of isolations from a methanolic extraction of *E. purpurea* were carried out to yield [1] a fraction containing an alkamides mixture [2], dodeca-2E,4E,8Z,10Z(E)-tetraenoic acid isobutylamide, and [3] cichoric acid. Alkamide mixture dose: 5 and 25 μg/mL Dodeca-2E,4E,8Z,10Z(E)-tetraenoic acid isobutylamide dose: 5 and 100 μM Cichoric acid dose: 50 and 100 μM DURATION: 4 and 20 h	Negative control: no treatment and no stimulation Positive control: stimulation with no treatment	Decreased secretion IL- 1β, IL-6, IL-10, IL-12p70, IL-13, IL-1α and IL-2, MCP-1, MIP-1β9, RANTES and GM-CSF	1
Hwang, 2004 [85]	Presented in part during receipt of the ‘‘Paul E. Strandjord Young Investigator Award for 2003″, at the 38th annual meeting of the Academy of Clinical Laboratory Physicians and Scientists (ACLPS), Tucson, AZ (June 2003).	Female BALB/c mouse splenocytes, further sub fractionated to adherent and non-adherent cell populations	N/A	*E. purpurea*	Liquid extract: fresh *Echinacea* root juice, mature seed, fresh leaf juice and fresh fruit juice extracted in 44–50% alcohol Solid extract: solid extract (dried *Echinacea* root and leaf) dissolved in either in distilled water or absolute alcohol in the ratio of 25 mg of solid extract per ml of solvent Dose of *Echinacea* preparation: 1 mg/mL DURATION: 48 h	None	Increased production: IL-6, IL-10, MIP-1α and TNF-α No change in production: IFN-γ, IL-1β, IL-2 and IL-12	3
Kapai, 2011 [86]	N.N. Blokhin Russian Oncological Research Center, the Russian Academy of Medical Sciences, Moscow	MNL isolated from heprin-stabilized periphereal blood	N/A	*E. purpurea t*incture	*E. purpurea* tincture in a series of 10-fold dilutions. the active concentration was D1-D17. DURATION: 48 h	Saline containing EtOH	Increased production: IL-1, IL-8, IL-1β, IL-10 and IL-14	3
Lee, 2015 [87]	National Research Foundation of Korea (NRF)funded by the Ministry of Education (NRF-2014R1A1A2008663).	HMC-1	PMACI A23187	Chicoric acid	≥95% purity Dose: 12.5, 25, or 50 μM DURATION: 24 h	Negative control: no treatment and no PMACI stimulation Positive control: no treatment and PMACI stimulation	Decreased mRNA expression: IL-6, IL-1β and TNF-α	1
Li, 2017 [88]	Grants from the National Natural Science Foundation of China (No. 31472128).	Bone marrow-derived dendritic cells from C57BL/6 mice	LPS (50 ng/mL)	*E. purpurea*	Extract purchased from Shandong Qilu Animal Health Co., Ltd. Chemical composition of extract: cichoric acid (3.045%), caftaric acid (1.575%), chlorogenic acid(0.065%), dodeca-2E, 4E, 8Z, 10E/Z-tetraenoic acid isobutylamide(1.635%). Dose: 400 μg/mL DURATION: 24 h	Negative control: no treatment	Increased secretion: IFN-γ, IL-10 and IL-12	1
Luettig, 1989 [89]	Not stated	Spleen T cells, thioglycolate-induced peritoneal macrophages, bone marrow macrophages, and resident peritoneal macrophages from C57BL/6 mice	T Cells - ConA at 1 and 5 μg/mL B cells - LPS 50 μg/mL Macrophages in virto - LPS 100 μg/mL	Arabinogalactan from *E. purpurea*	Varied per experiment, but ranged from 3.7 to 500 μg/mL DURATION: 18–48 h	Negative control: no treatment Positive control: LPS (10 or 20 μg/mL)	Increased production: IFN-β2, IL-1 and TNF-α No change in production: IL-2	3
Matthias, 2007 [90]	MediHerb Research Laboratories, Queensland, Australia	Mouse macrophage cell line	LPS (0.1 μg/mL) or PMA (2 nM)	Alkylamide 1. (2E)-N-isobutylundeca-2-ene-8,10-diynamide; Alkylamide 2. (2E,4E,8Z,10Z)-N-isobutyldodeca-2,4,8,10-tetraenamide.; An ethanolic extract (*Echinacea* Premium Liquid; EPL) of *E. purpurea* (300 mg/mL), *E. angustifolia* (200 mg/mL) roots and EPL alkylamide fraction (EPL AA) was separated from caffeic acid fraction and cichoric acid	Alkylamides concentration 0.2 ng/mL; cichoric acid concentration 0.8 ng/mL DURATION: 4 and 20 h	Unstimulated cells	Decreased production: TNF-α	3
McCann, 2007 [91]	Grant P01ES012020 from the National Institute of Environmental Health Sciences (NIEHS) and the Office of Dietary Supplements (ODS), NIH.	Human peripheral blood mononuclear cells (isolated from 19 subjects between the ages of 19 and 36 who donated blood 8 h pre- and 4 weeks post- receiving the 2005/2006 trivalent influenza Fluzone vaccine)	Influenza type A H1N1 virus (A/New Caledonia/20/99)	*E. angustifolia, E. pallida, E. paradoxa, E. purpurea, E. sanguinea, E. simulata, and E. tennesseensis*	Root tinctures of each species extracted in 50% EtOH/50% water at a ratio of 1 part plant/9 part solvent. Tinctures were stored at −20 °C for 24 months. Dose: 1:12.5 dilution DURATION: 24 or 48 h	Experiment 1: Negative control: no treatment Experiment 2: Negative control: no treatment on uninfected cells Positive control: no treatment on infected cells	Increased levels: IL-10 Decreased levels: IL-2 No change in levels: IFN-γ, IL-12 and TNF-α	1
Mishima, 2004 [92]	NAGARAGAWA Research Center, Suxuka University of Medical Science Graduate School of Health Science	Peripheral blood cells and T lymphocytes	Radiation	*E. purpurea*	360 mg/kg; mice administered treatment every other day every other day DURATION: 3 weeks	Blood from; Mice + saline/no *E.Purpurea + radiation*, Mice + *E.Purpurea + no radiation,* Mice + radiation only	Increased production: IFN-γ	1
Moazami, 2015 [93]	Partially funded by NC State's Office of Research, Innovation, and Economic Development, in partnership with the Kenan Institute for Engineering, Technology and Science and the Center for Comparative Medicine and Translational Research.	Murine RAW 264.7 macrophage-like cells	LPS (10 ng/mL)	Fatty acid amide dodeca-2E,4E-dienoic acid isobutylamide, a constituent of *E. purpurea*, and a series of analogs that varied by unsaturation, alkyl chain length, and amide head group	Fatty acid amide was chemically synthesized de novo, and analogs were created by altering the double bonds and/or the alkyl chain length in the fatty acid unit. Dose: 100 μM DURATION: 18 h	Negative control: treatment without LPS stimulation Positive control: LPS stimulation without treatment	Decreased production: TNF-α	1
Morazzoni, 2005 [94]	Dipartimento di Scienze Cliniche e Biologiche, Università degli Studi di Torino, Torino, Italy	J774. a murine macrophage cell	LPS (1 μg/mL)	*E. angustifolia*	The roots were exhaustively treated with 90% EtOH for echinacoside extraction and then counter- extracted with n-hexane for isobutylamides elimination. Wet roots were extracted with 15% aq. DURATION: 7 days	Negative control: no treatment	Increased production: IFN-γ	1
Olah, 2017 [95]	Bundesministerium für Wirtschaft und Energie (BMWi), Germany (ZIM-KOOP, grant number: KF2611301MD0; Dr. August Wolff GmbH & Co. KG Arzneimittel (Bielefeld, Germany); Hungarian research grants (NRDIO 121360, NRDIO 120552).	Human immortalized HaCaT keratinocytes	Polyinosinic-polycytidylic acid	*E. purpurea* root extract	Extract is prepared by supercritical CO2-extraction of *E. purpurea* roots. Dose: 20 μg/mL DURATION: 3 and 24 h	Negative control: no treatment and no stimulation Positive control: stimulation with no treatment	Decreased mRNA expression: IL-6 and IL-8	1
Pomari, 2014 [96]	Progetto Nutriheart POR FESR 2007–2013 Friuli Venezia Giulia, Italy.	RAW264.7 murine macrophages	H_2_O_2_ (200 μM)	*E. angustifolia*	Commercial ethanolic root extract standardized to ≥4% echinacoside Dose: 10 μg/mL DURATION: 24 h	Negative control: no treatment and no stimulation Positive control: stimulation with no treatment	Increased mRNA expression: TNF-α Decreased mRNA expression: IL-1β	1
Pugh, 2004 [97]	National Center for Natural Products Research, University of Mississippi, University,	THP-1 human monocyte cell line	LPS (10 μg/mL)	*E. angustifolia, E. pallida* and *E. purpurea* - specifically melanin extracted from the latter plants	0.1, 0.4 and 1.0 μg/mL DURATION: 4 days	Negative control: no treatment	Increased secretion: IL-1β	1
Raduner, 2006 [98]	Initial financial support provided by Prof. Dr. Jorg Heilmann	Human peripheral whole blood [from healthy volunteers]	LPS (313 ng/mL)	3 alkylamides from *E. purpurea*: A1 (dodeca-2E,4E,8Z,10Z-tetraenoic acid isobutylamide), A2 (dodeca-2E,4E-dienoic acid isobutylamide), and A3 (undeca-2E-en-8,10-diynoic acid isobutylamide).	A2 was isolated from *E. purpurea*. A1 and A3 were gifted by MediHerb, Australia. Dose: 5 nM, 50 nM, 500 nM, and 5000 nM DURATION: 18 h	Negative control: treatment without stimulation Positive control: stimulation without treatment	Decreased expression: IL-1β, IL-6, IL-8, IL-10, IL-12p70 and TNF-α	1
Randolph, 2003 [37]	Nutrilite Health Institute, Access Business Group, LLC, Buena Park, California and Source Precision Medicine, Boulderm Colorado	THP-1 human monocyte cell line	18S mRNA	*E. angustifolia root, E. purpurea* root and herb	10 μg/mL, 50 μg/mL, 250 μg/mL DURATION: 6 h	Untreated cells	Increased gene expression: IL-1α, IL1β, IL-8, IL-10 and TNF-α	3
Rininger, 2000 [99]	Paracelsian, Incorporated, Ithaca, New York	RAW264.7 macrophage cells	LPS 0.1 μg/mL	*E. purpurea*	5 μg/mL, 20 μg/mL, 80 μg/mL, 320 μg/mL DURATION: 48 h	Medium alone and LPS + medium	Increased production: IL-1α, IL-1β, IL-6, IL-10 and TNF-α	1
Ritchie, 2011 [100]	Founded by A. Vogel Bioforce AG, Switzerland; Funded by Bioforce, Switzerland.	Blood samples	Zymosan (333 μg/mL) or LPS (from *E.Coli* at 100 ng/mL)/super-antigen SEB at 25 ng/mL)	*E. purpurea*	Echinaforce *-* patient took 4 1 mL doses for 5 days, then 10 1 mL doses for 3 days. Blood sample taken each day for analysis; Echinaforce phytochemical profile: 264.4 μg/mL caftaric acid, 40.2 μg/mL chlorogenic acid, 313.8 μg/L cichoric acid, 6.9 μg/mL echinacoside, 35.9 μg/mL dodeca tetraene; Echinaforce made from freshly harvested herbs and roots of *E. purpurea* in a 95:5 ratio. DURATION: 8 days of supplementation, blood cells stimulated for 24 h	Baseline - blood samples prior to Echinaforce supplementation	Increased production: IFN-γ, IL-8 and IL-10 Decreased production: IL1-β and TNF-α	3
Sasagawa, 2006 [101]	Bastyr Univerisity, Department of Basic Sciences, Kenmore, United States	Jurakat cells	PHA and PMA; Treatments: PHA; 10 ng/mL PMA; or 1 μg/mL PHA+1 ng/mL PMA	*E.purpurea* extract*,* Alkylamides (1. Dodeca-2(E),4(E),8(Z),10(Z)-tetraenoic acid isobutylamide; 2. Dodeca-2(E),4(E)-dienoic acid isobutylamide in 05% EtOH) and caffeic acid derivatives (3. Caftaric acid 47.5% EtOH; 4. Cichoric acid in 95% EtOH; 5. Chlorogenic acid 47.5% EtOH)	*E.purpurea* extract; 0.1 μg/mL, 1 μg/mL, 10 μg/mL, 50 μg/mL and 100 μg/mL in 95:5, 75:25, 50:50, 25:75 EtOH:water mixtures.//*Echinacea* consitituents*;* stock concentration of 5 mg/mL diluted to final concentration of 0.625–25 μg/mL DURATION: 24 h	0.5% EtOH vehicle	Decreased production: IL-2	1
Senchina, 2005 [102]	Grant number P01ES012020 from the National Institute of Environmental Health Sciences (NIEHS) and the Office of Dietary Supplements (ODS), NIH.	Human monocytes [isolated from blood from 5 healthy human donors]	N/A	*E. angustifolia* var. *angustifolia, E. pallida, E. purpurea, E. sanguinea, and E. tennesseensis*	3 extracts for each *Echinacea* species: 50% EtOH, cold water infusion, and hot water infusion [1 part plant to 9 parts solvent]. Extracts were stored at 4 °C and tested at 1 and 4 days post-extraction. Dose not stated. DURATION: 24 h	Negative control: no treatment	Increased production: IL-10 (immediately), IL-12, TNF-α Decreased production: IL-10 (later time point)	3
Senchina, 2006 [103]	Grant number P01ES012020 from the National Institute of Environmental Health Sciences (NIEHS) and the Office of Dietary Supplements (ODS), NIH.	Human peripheral blood mononuclear cells (from 15 healthy human young adult donors)	N/A	*E. angustifolia, E. pallida, E. paradoxa, E. purpurea, E. sanguinea, E. simulata, and E. tennesseensis*	Method of extraction not stated. Extracts were stored at −20 °C for 1 month before beginning experiments. Dose not stated. DURATION: 24 h	Negative control: no treatment	Increased production: IL-1β and TNF-α No change in production: IL-2	3
Senchina, 2006 [104]	Grant number P01ES012020 from the National Institute of Environmental Health Sciences(NIEHS) and the Office of Dietary Supplements (ODS), NIH	Human peripheral blood mononuclear cells (isolated from older adults 6 months post receiving trivalent influenza vaccine)	Influenza A/New Caledonia/20/99 (H1N1) virus or the Influenza A/Wyoming/03/2003 (H3N2) virus	*E. angustifolia, E. pallida, E. paradoxa, E. purpurea, E. sanguinea, E. simulata, and E. tennesseensis*	50% ethanolic tinctures of roots from each species [1 part plant, 9 parts solvent]. Dose: 1:12.5 dilution DURATION: 48 h	Negative control: no treatment on infected cells	Increased levels: IL-10 Decreased levels: IL-2 and IFN-γ	1
Senchina, 2009 [105]	Grant Number P01ES012020 from the National Institute of Environmental Health Sciences (NIEHS) and the Office of Dietary Supplements (ODS), NIH.	Human peripheral blood mononuclear cells (from 16 subjects between the ages of 19 and 36 who donated blood)	N/A	*E. tennesseensis*	Separate 50% EtOH tinctures prepared from roots, stems, leaves, and flower. Tincture aliquots were stored at three different temperatures (4, −20, and −80 °C) for 21 h before testing. The −20 °C aliquots were saved and tested again 1 month later. Dose: 1:12.5 dilution DURATION: 24 h	Negative control: no treatment	Increased production: IL-1β, IL-10 and TNF-α No change in production: IL-2	1
Senchina, 2009 [106]	faculty start-up funds allocated to DSS at Drake University.	Human blood mononuclear cells (from 12 healthy young men)	2 separate exercise bouts [1]: VO2max test and [2] 90 min of cycling at 85% of ventilatory threshold	*E. tennesseensis*	Separate 50% EtOH tinctures prepared from roots and flowers. Extracts were stored at−80 °C undisturbed for 3 years before the study took place. Dose: 50 μL DURATION: 24, 48 and 72 h	Negative control: no exercise stimulation and no treatment Positive control: exercise stimulation with no treatment	No change: IL-1β, IL-10 and TNF-α	1
Senchina, 2010 [107]	grant number P01Es012020 from NIEHS and the Office of Dietary Supplements.	RAW264.7 murine macrophage cells	HSV-1 virus	*E. angustifolia* var. *strigosa, E. purpurea, and E. tennesseensis*	3 separate tinctures of dried root samples of the three species made with 50% EtOH/50% water at a ratio of 1:9 parts plant material:solvent. *E. purpurea* roots were also made into a 4th extract with 95% EtOH and using the Soxhlet apparatus. Dose: 1:12.5 dilution DURATION: 24 h	Negative control: EtOH at the same concentration (<0.2%) Positive control: Poly I:C	Decreased levels: IFN-α No Change in levels: IFN-β	1
Senchina, 2011 [108]	faculty start-up funds given to DSS at Drake University.	Human peripheral blood mononuclear cells [from 16 subjects (9 males, 7 females, age 23.5 ± 3.8 years) who donated blood]	LPS and PHA antigen	*E. laevigata, E. angustifolia, E. pallida, and E. purpurea*	Root tinctures of each species extracted in 50% EtOH/50% cell culture water at a ratio of 1:9 parts plant material:solvent. Dose: 50 μL/well DURATION: 24, 48 or 72 h	Negative control: no treatment Positive control: LPS and PMA antigen	Increased levels: IL-10 and TNF-α No change in levels: IL-2	1
Sharma, 2006 [109]	Not stated	The tracheo-bronchial line BEAS-2B and the rhinovirus-sensitive H-1 derivative of HeLa cells	Rhinovirus type 14	*E. purpurea*	Two extracts: E1: an expressed juice extract of the aerial parts of *E. purpurea* E2: a 50% alcoholic tincture, derived from *E. purpurea* roots (1:9 w/v) Dose: 100 μg/mL of E1 or 50 μg/mL of E2 DURATION: 24–96 h	Negative control: no treatment on uninfected cells Positive control: no treatment on virally infected cells	Increased secretion: IL-1β, IL-2, IL-3, and IL-7 Decreased secretion: IFN-γ, IL-1⍺, IL-1β, IL-2, IL-3, IL-5, IL-6, IL-7, IL-8, IL-15, IL-17, TNF-α, GM-CSF, CCL8, CCL10, CCL11, MIP-1α, MIP1β and MIP-4	3
Sharma, 2009 [110]	Not stated	The tracheo-bronchial line BEAS-2B, H-1 sub clone of HeLa cells, the lung-derived epithelial cell line A549, and human skin fibroblasts	Rhinovirus types 1A and 14	*E. purpurea*	Echinaforce by A. Vogel Bioforce AG, Switzerland: a 65% ethanol extract of freshly harvested aerial parts supplemented with 5% roots. Dose: dilutions of 1:20, 1:100, 1:200, and 1:400 DURATION: 48 h	Negative control: no treatment on uninfected cells Positive control: no treatment on virally infected cells	Decreased secretion: IL-6 and IL-8	3
Sharma, 2009 [111]	Not stated	Two human epithelial cell lines: the tracheo-bronchial line BEAS-2B and the lung-derived epithelial cell line A549 as well as human skin fibroblasts	Viruses: RV1A, RV14, influenza, RSV, adenovirus types 3 and 11, and HSV	*E. purpurea*	Echinaforce obtained from A. Vogel Bioforce AG, Roggwil, Switzerland, batch no.: 018451: standardized preparation derived by EtOH extraction of freshly harvested *E. purpurea* herb and roots(95:5) Dose: 1:100 dilution of *Echinacea* in DMEM without serum, corresponding to a final concentration of 160 μg/mL (dry mass/vol) DURATION: 24 and 48 h	Negative control: no treatment on uninfected cells Positive control: no treatment on virally infected cells	Decreased levels: IL1-α, IL-1β, IL-5, IL-6, IL-8, MIP-1α, MIP-1β, GRO-α, MCP-1, CCL5 and TNF-α	3
Sharma, 2010 [112]	Not stated	A total of three, separate, normal human airway epithelial tissues (code AIR-100), from three different donors	Rhinovirus type 1A	*E. purpurea*	Echinaforce by A. Vogel Bioforce AG, Switzerland: a 65% EtOH extract of freshly harvested aerial parts supplemented with 5% roots. Dose: 1:100 dilution of Echinaforce DURATION: 24 and 48 h	Negative control: no treatment on uninfected cells Positive control: no treatment on virally infected cells	Decreased secretion: IL-6 and IL-8	1
Sharma, 2010 [113]	Not stated	Two human epithelial cell lines: the tracheo-bronchial line BEAS-2B and the lung-derived epithelial cell line A549 as well as human skin fibroblasts	*H. influenzae* *L. pneumophila* MSSA MRSA S. pyogenes	*E. purpurea*	Echinaforce by A. Vogel Bioforce AG, Switzerland: a 65% EtOH extract of freshly harvested aerial parts supplemented with 5% roots. Dose: 1:100 dilution of *Echinacea* in DMEM without serum, corresponding to a final concentration of 160 μg/mL (dry mass/vol) DURATION: 48 h	Negative control: no treatment on uninfected cells Positive control: no treatment on virally infected cells	Decreased secretion: IL-4, IL-6 and IL-8, MIP-1α, GRO-α, MCP-1 and GM-CSF	3
Sharma, 2011 [114]	Not stated	Two human epithelial cell lines: the tracheo-bronchial line BEAS-2B and the lung-derived epithelial cell line A549 as well as human skin fibroblasts	Propionibacterium acnes	*E. purpurea*	Echinaforce by A. Vogel Bioforce AG, Switzerland: a 65% EtOH extract of freshly harvested aerial parts (drug extract ratio 1:12) supplemented with 5% roots (drug extract ratio 1:11). Dose: 1:100 dilution of *Echinacea* in DMEM without serum, corresponding to a final concentration of 160 μg/mL (dry mass/vol) DURATION: 48 h	Negative control: no treatment on uninfected cells Positive control: no treatment on infected cells	Decreased secretion: IL-6, IL-8 and TNF-α	3
Spelman, 2009 [115]	University of North Carolina Greensboro, Department of Chemistry and Biochemistry, Greensboro, United States	Jurakat T cells	PMA (1.25 ng/mL) or PHA (0.25 ng/mL)	*E. angustifolia*-derived alkylamide undeca-2E-ene-8,10-diyonic acid isobutylamide (This chemical constituent binds to PPAR-γ receptor to inhibit IL-2 production thus researchers explored this).	0.033 μg/mL, 0.1 μg/mL, 0.33 μg/mL, 1 μg/mL, 3.3 μg/mL DURATION: 18 h	EtOH/DMSO vehicle	Decreased secretion: IL-2	1
Stimpel, 1984 [116]	Not stated	Bone marrow macrophages from C57BL/10 mice	100 μg of LPS or μg of EPS	Purified polysaccharides from *E. purpurea*	Polysaccharides were purified by chromatography from alkaline-water extracts of *E. purpurea.* Dose: 100 μg DURATION: 8–24 h	Negative control: unstimulated macrophages Positive control: LPS (10 μg)	Increased production: IL-1	3
Sullivan, 2008 [117]	Natural Sciences and Engineering Research Council of Canada and the Nova Scotia Health Research Foundation, Halifax, Nova Scotia, Canada.	Murine peritoneal macrophages	LPS	*E. purpurea; IL-6* 2400, 1200, 600, 300 and 150 μg/mL//IL-12, IL-1B 500 μg/mL	IL-6 48 h//IL-12, IL-1B 24 h. DURATION: 24 or 48 h	IL-6 LPS positive control and media and negative control//IL-12, IL1B media control	Increased production: IL-6 and IL-12, TNF-α No change: IL-1β	
Todd, 2015 [118]	Grant #1R15AT007259 from the National Centre for Complementary and Alternative Medicine, Maryland, United States.	RAW 264.7 macrophage-like cells	LPS 100 μg/mL	75% *Echinacea* extract (ground root), various liquid partitions, EE, HL, ML, WL and CL (Each of these fall under one of the fractions 1–13, see Fig. 1)	TNF 50 μg/mL, 100 mg/mL//Chemokines - varying degrees of alkylamides for fractions 1–13 and CL (precise concentrations and chemical structures in paper, Table 1 and Fig. 3) DURATION: 16–18 h	Medium	Decreased production: CCL3, CCL5 and TNF-α	3
Vimalanathan, 2009 [119]	Not stated	BEAS-2B	Rhinovirus type 14 (RV 14) (infection at 1 virus/cell (1 pfu/cell))	Root, leaf and flower extracts of *E. purpurea* (L.) Moench, Root extracts of *E. angustifolia* (D.C.) and *E. pallida* (Nutt.) Nutt.	250 μg/mL DURATION: 48 h	Cells with no virus + treatment	Decreased production: IL-6 and IL-8	3
Vimalanathan, 2017 [120]	A.Vogel Bioforce AG, Roggwill(TG), Switzerland	BEAS-2B	Influenza (H3N2) and bacterial LPS	Echinaforce (E. purpurea*)*	CFU assay - 1:200 (50 μg/mL), 1:400 (40 μg/mL), 1:800 (20 μg/mL)//Cytokine assay - 1:100, 1:200, 1:400//NFκB p65 expression assay - 1:200, 1:400 DURATION: 24 and 48 h	CFU assay, cytokine assay, NFκB expression assay - vehicle alone, no treatment	Decreased production: IL-6 and IL-8	1
Wang, 2006 [121]	Agricultural Biotechnology Research Center, Academia Sinica, Nankang, Taipei 115, Taiwan, Republic of China	Human DCs	LPS (1 μg/mL)	*E. purpurea* - stem + leaf (0.10% alkylamide) and root (3.01% alkylamide)	Used 100 μg/mL for data presented DURATION: 4 and 16 h	Vehicle control	Increased gene expression: IL-7, CCL2 and CCL4 Decreased gene expression: IL-1β, CCL3 and CCL8	1
Wang, 2008 [122]	Agricultural Biotechnology Research Center, Taiwan	Human immature dendritic cells	LPS (100 ng/mL)	*E.Purpurea -* Stem and leaf fractions in *n*-butanol (BF/S + L/Ep) or cichoric acid	Concentration of cichoric acid 8.4% w/w and rutin 22.3% w/w DURATION: 4 and 24 h	0.1% DMSO as vehicle control	Increased gene expression: IL-1β, IL-8, IL-18, CXCL1, CCL2 and CCL5 Decreased gene expression: IFN-α	1
Wilasrusmee, 2002 [123]	Not stated	Human peripheral blood mononuclear cells	5000-rad γ -irradiated stimulator cells	*E. purpurea*	Dried and ground fresh herb homogenized in RPMI and filtered. Dose not specified. DURATION: 5 days	Negative control: no treatment	No change in production: IL-2 and IL-10	3
Woelkart, 2006 [124]	Institute of pharmaceutical sciences, department of pharmacognosy	Blood samples	LPS 100 pg mL + E51:F51	*E.purpurea* tincture (Echinaforce*)* or tablet	*E. purpurea* tincture containing 0.018 mg/mL of dodeca-2E,4E,8Z,10E/Z-tetraenoic acid isobutylamides and 1 *E.purpurea* tablet is 0.006 mg DURATION: 23 h	Alcohol or lactose	Decreased production: IL-8 and TNF-α No change in production: IL-6	3
Wu, 2009 [125]	PolinaceaTM was donated by Indena s.p.a.; MiUR (PRIN 05) and Università degli Studi della Tuscia, and the Asia Link Project ‘‘Organic Farming: ethical, economic, technical and scientific aspects in a global perspective	Peripheral blood mononuclear cells (from six healthy Holstein heifers)	ConA (1 μg/mL)	*E. angustifolia*	Hydroethanolic root extract called Polinacea donated by Indena s.p.a. (Settala, Milan, Italy). Doses: 0, 6.3, 20, 60, and 180 μg/mL DURATION: 72 h	Negative control: no stimulation and no treatment	No change in secretion: IFN-γ	3
Yang, 2018 [126]	State Key Laboratory for Conservation and Utilization of Subtropical Agro-Bioresources, South China Agricultural University	Spleen lymphocytes	ConA (100 μg/mL)	Tetraploid (CPE4) (85.51% crude polysaccharide) and diploid (CPE2) *E. purpurea* (44.65% crude polysaccharide)	0.5–0.0039 mg/mL DURATION: 48 h	10 *μ*g/mL ConA	Increased production: IFN-γ, IL-2, TNF-α	3
Yao, 2019 [127]	College of Veterinary Medicine, South China Agricultural University	Chicken bone marrow-derived dendritic cells	5 μg/mL LPS	*E. purpurea* polysaccharide (EPP) and sulfated EPP (sEPP)	EPP (2−2, 2–3, 2–4 mg/mL, marked as EPPH, EPPM, EPPL, respectively) or sEPP (2–7, 2–8, 2–9 mg/mL, marked as sEPPH, sEPPM, sEPPL, respectively) DURATION: 48 h	Serum-free DMEM and only LPS stimulation	Increased production: IFN-γ, IL-2 Decreased production: IL-4 and IL-10	3
Zhai, 2007 [128]	the National Institute of Environmental Health Sciences (grant P01ESO12020) and the Office of Dietary Supplements, National Institutes of Health.	Splenocytes	ConA of 1 and 3 μg/mL and LPS (10 μg/mL)	*E.angustifolia, E.pallida,* and *E.purpurea*	130 mg/kg delivered orally DURATION: 7 days	Vehicle control: 5% EtOH	Decreased secretion: TNF-α No change in secretion: IL-1β and IL-10	1
Zhang, 2012 [129]	grant number 9P50AT004155-06 from the National Center for Complementary and Alternative Medicine (NCCAM) and the Office of Dietary Supplements (ODS), National Institutes of Health (NIH).	RAW264.7 mouse macrophage cells	LPS (1 μg/mL)	*E. angustifolia, E. pallida, E. paradoxa, E. paradoxa* var. *paradoxa*, and *E. purpurea* Bauer ketones 22, 23 and 24	*E. paradoxa* var. *paradoxa* was fractionated into 5 fractions by semipreparative HPLC system. Doses: 184 μg/mL (fraction 1), 75 μg/mL (fraction 2), 101 and 20 μg/mL (fraction 3), 20 and 3.2 μg/mL (fraction 4), 36 and 20 μg/mL (fraction 5), 187 and 20 μg/mL (fraction 6). Bauer ketones 22, 23 and 24 (present in fraction 5) where chemically synthesized. Doses: 3.1 μM (#22), 1.6 μM (#23), and 9.7 μM (#24). DURATION: 24 h	Negative control: stimulation with no treatment Positive control: quercetin	Decreased production: IL-1β, IL-6 and TNF-α	1

BEAS-2B: Human Bronchial Epithelial Cell Line; ConA: Concanavalin A; CXCL/CCL: Chemokine Ligand; CL: Chloroform Layer; DC: Dendritic Cells; DMEM: Dulbecco's Modified Eagle Medium; DMSO: Dimethylsulfoxide; EE: Ethanol Extract; EPP: *E. purpurea P*olysaccharide; EPS: Extracellular Polymeric Substances; EtOH: Ethanol; g: Gram; GM-CSF: Granulocyte-macrophage Colony-stimulating Factor; GRO: Growth Regulated Oncogene; HaCaT cells: Human Keratinocyte Cells; HL: Hexane Layer; HMC-1: Human Mast Cells; H_2_O_2_: Hydrogen Peroxide; IFN: Interferon; Il: Interleukin; kg: Kilogram; LPS: Lipopolysaccharide; MCP: Monocyte Chemoattractant Protein; MIP: Macrophage Inflammatory Protein; ml: Millilitre; ML: Methane Layer; MNL: Mononuclear Leukocyte; MRSA: Methicillin-resistant Staphylococcus Aureus; MSSA: Methicillin-susceptible Staphylococcus Aureus; NADPH: Nicotinamide adenine dinucleotide phosphate; NFκB: Nuclear Factor kappa B; ng: Nanogram; NK: Natural Killer; nM: Nanomolar; OVA-FITC: Ovalbumin Fluorescein Conjugate; PHA: Phytohemagglutinin; PMA: Phorbol 12-myristate 13- acetate; PMACI: Phorbol-12-myristate 13-acetate plus calcium ionophore; PPAR-γ: Peroxisome Proliferator-activated Receptor gamma; RANTES**:** Regulated on Activation Normal T Expressed and Secreted; RBL: Rat Basophilic Leukemia cells; RPMI: Roswell Park Memorial Institute Medium; SEB: Staphylococcal enterotoxin B; sEPP: Sulfated *E. purpurea* Polysaccharide; TNF: Tumour Necrosis Factor; TPH-1: Tryptophan hydroxylase-1; μg: Microgram; μM: Micrometre; WL: Water Layer.

a 1 = reliable without restrictions, 3 = unreliable.

The most commonly studied *Echinacea* species in human, animal and *in vitro*/*ex vivo* studies alike was E. purpurea. Approximately 66% of all studies used E. purpurea alone and another 19% used E. purpurea in combination with other species. The second most commonly studied species was E. angustifolia; with approximately 8% of studies using it on its own and 18% using it in combination with other species.

Human studies were conducted primarily in the USA (38%, n = 5), followed by Italy and Germany (23%, n = 3 each), Indonesia (8%, n = 1) and Ukraine (8%, n = 1). Of the 13 human studies, eight (61%) examined the effects of *Echinacea* on healthy adults. The remaining five studies examined the effects of *Echinacea* on: healthy male triathletes training for competition [34], healthy adults exposed to rhinovirus [30], teenagers and adults with new inset of the common cold [28], adults in clinical remission of chronic herpes [35], and COPD outpatients [29]. The largest human study was a clinical trial with 713 participants [28] and the smallest were two non-randomized studies without a control group [39,40] with six participants each. The average number of participants in human studies was 112 (SD = 208) and the median was 40. The *Echinacea* dosage and duration of treatment employed also varied widely, ranging from a one-time injection containing 5 mg of *Echinacea* polysaccharides [36] to a daily dose of 8000 mg of *Echinacea* capsules for 28 consecutive days [32]. A total of four studies [31,32,34,37] implemented 28-day interventions and three employed a one-time dose [36,38,40]. Concerningly, two studies [33,35] did not specify the dosage of *Echinacea* used. Moreover, *Echinacea* tablets or soft gel capsules were the most common type of intervention. Additional interventions included *Echinacea* lozenges, syrup, juice and tinctures. All of the human studies except for one [31] assessed changes in interleukins, with IL-6 being the most common, closely followed by IL-8, IL-1B, then IL-10, IL-2, IL-12 and IL-3. The second most commonly studied cytokine was TNF (61%, n = 8). Lastly, three studies (23%) assessed changes in INF and only one (8%) assessed changes in GM-CSF. None of the human studies included assessed changes in chemokines.

Animal studies were conducted in mouse or rat models, although studies also included dogs [54], tilapia [45], and guinea pigs [55]. Sixteen trials had a duration of at least two weeks while five lasted four to seven days [41,43,50,57,131] and three lasted one day or less [60,61,63]. The daily dose of *Echinacea* varied widely from 5 to 500 mg/kg per day.

The cell culture studies used a variety of immune cells. Immune stimulation was achieved through a variety of methods; the most common where exposure to LPS (n = 29), viruses (n = 14) and phytohemagglutinin and/or phorbol 12-myristate 13- acetate (n = 10). Studies assessed changes in the amount of cytokines produced or changes in genetic expression following exposure to *Echinacea*.

### 3.1 Change in cytokine levels

**Fig. 2:**
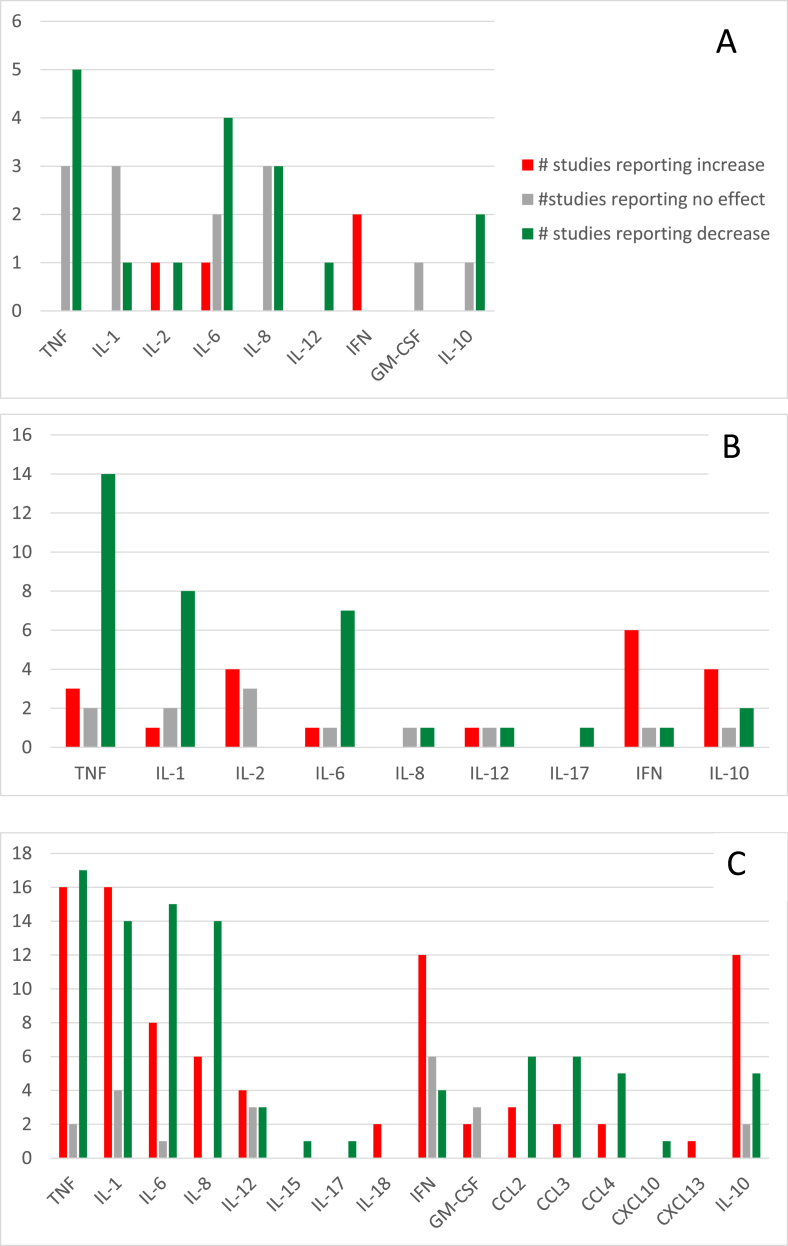
Change in cytokine levels following Echinacea exposure. A: Human studies, B: animal Studies, C: Cell culture studies.

The changes in cytokine levels that followed E*chinacea* supplementation are presented in Fig. 2. Results are presented for the cytokines relevant to the progression of cytokine storm. Among the human studies, decreased levels of the pro-inflammatory cytokine IL-6, IL-8, and TNF were reported by 57, 50, and 62% of studies that measured these cytokines, respectively. Among the animal studies decreased levels of pro-inflammatory cytokines IL-1, IL-6, and TNF, were reported by 73, 78, 74% of studies that measured these cytokines, respectively. However, increased levels of the pro-inflammatory cytokine IL-2 were reported by 57% of animal studies. In addition, an increase in levels of the anti-inflammatory cytokine IL-10 were reported by 57% of animal studies that measured this cytokine. Among the cell culture studies, decreased levels of pro-inflammatory cytokines IL-6, IL-8, CCL2, CCCL3, and CCL4 were reported by 63, 70, 67, 75, 71% of studies that measured these cytokines, respectively. Moreover, nearly two thirds of the cell culture studies that measured levels of the anti-inflammatory cytokine IL-10 reported an increase. IFN levels were increased in the majority of human, animal, and cell culture studies; while this cytokine is considered to be pro-inflammatory, decreased levels of IFN have been detected among COVID-19 patients. None of the studies reported cases of cytokine storm.

### 3.2 Risk of bias assessment

**Fig. 3:**
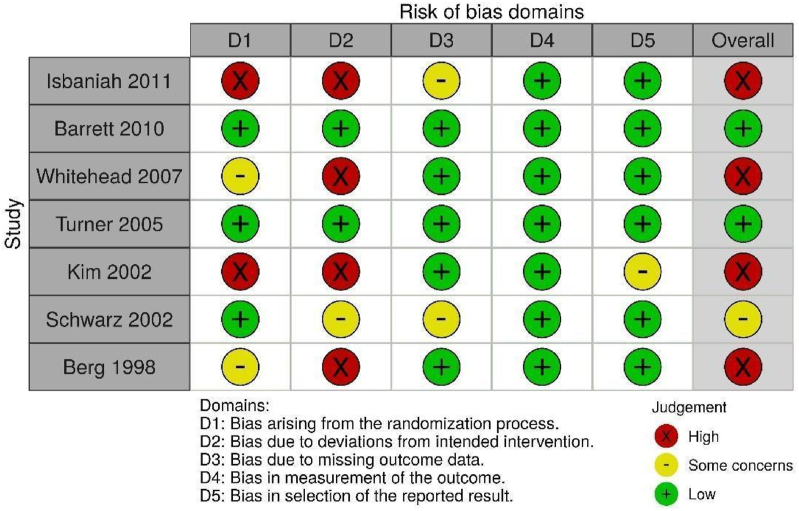
Risk of Bias 2.0 for human randomized controlled trials.

**Fig. 4:**
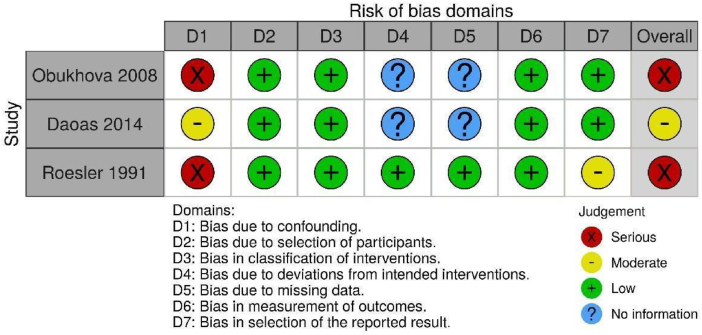
ROBINS-I Assessment of bias for non-randomized human studies with a comparison.

The results of the risk of bias assessments for the human RCT and non-RCT studies are presented in Fig. 3, Fig. 4. In total, six of these studies had a “high risk of bias”, two studies had “some concerns” or “moderate risk of bias” and two studies had “low risk of bias”. Among the pre-post human studies, two received a rating of “fair” and one received a rating of “poor”. Among the animal studies, each one received a rating of “probably high risk of bias” in at least one category. Three received a rating of “definitely high risk of bias” in one category. Additional information on the risk of bias assessment for the pre-post and animal studies is found in Supplemental File 2. Among the cell culture studies, thirty-eight (55%) received as score of 1 corresponding to “reliable without restrictions”. Thirty-one (45%) received a score of 3 corresponding to “unreliable”.

## 4 Discussion

The present systematic review identified all human, animal, and cell culture data reporting the impact of *Echinacea* supplementation on cytokine levels. The data suggest that *Echinacea* supplementation may be associated with a decrease in the pro-inflammatory cytokines IL-6, IL-8 and TNF as well as an increase in the anti-inflammatory cytokine IL-10. In addition, it may be associated with an increase in IFN, a pro-inflammatory cytokine reported to be low in patients with COVID-19. Overall, the findings of the human and animal studies were more likely to report primarily anti-inflammatory effects. *Ex vivo* and *in vitro* studies demonstrated more of a mixture of pro- and anti-inflammatory effects; however, given that they were conducted in the isolation of cell culture rather than in the context of a highly complex, functioning immune system, the results may be less relevant to use in humans. The findings suggest that the use of *Echinacea* supplementation may be useful in the prevention or management of COVID-19-related cytokine storm in humans, however further targeted studies are needed.

Levels of IL-6 and TNF both independently predict COVID-19 disease severity and mortality [8] and may be important therapeutic targets. Therapies aimed at inhibiting these cytokines have demonstrated improvements in the clinical course of severely ill COVID-19 patients. A meta-analysis of studies administering the IL-6 receptor monoclonal antibody tocilizumab to patients with severe COVID-19 revealed a reduction in mortality and the need for mechanical ventilation [132]. The effects of other immunomodulatory agents including anakinra, an inhibitor of IL-1, and sarilumab and siltuximab, inhibitors of IL-6, were inconclusive [133]. Observational registry data from patients with inflammatory bowel disease who contracted COVID-19 suggest a possible benefit from taking anti-TNF medication in terms of a composite outcome of death or hospital admission, however not with either outcome alone [134]. A call to prioritize the study of anti-TNF therapy has been made [134]. Because IL-6 and TNF are independently associated with clinical outcomes, it has been hypothesized that therapy targeted at the inhibition of both cytokines simultaneously may yield additional benefit and warrant study [8]. *Echinacea* may decrease production of these two cytokines.

Among the studies identified in the present review, more studies reported an increase in IFN production than a decrease following *Echinacea* supplementation. While IFN-α and β are considered proinflammatory in nature, they also play a critical role in exerting an antiviral effect. Observation of depressed levels of IFN-α and β among COVID-19 patients has occurred [9]. While the trial reporting this finding was primarily cross-sectional, sequential assessment found that the depressed levels of IFN-α preceded worsening of disease severity and transfer to more intensive care [9]. The virus SARS-CoV, the causative agent of severe acute respiratory syndrome (SARS), inhibits production of IFNs in order to diminish the innate immune response of the host [135]. A need to explore therapeutic approaches to increase IFN in the treatment of COVID-19 has been proposed [9].

Additional evidence that may be considered regarding the potential usefulness of *Echinacea* in the management of COVID-19 include the herb's ability to decrease the severity and duration of acute respiratory tract infections [22] and *in vitro* data demonstrating direct antiviral effect of *Echinacea* against several coronaviruses including SARS-CoV-2([136]).

The present review has several strengths and limitations. Strengths of the review include a rigorous search strategy that was conducted in multiple databases, as well as duplicate screening and data extraction. The review process is limited by a high level of heterogeneity among the included studies and subsequently, the inability to complete meta-analysis. The findings are limited by the high risk of bias found in many of the included studies. They are also limited by the fact that none of the studies assessed the impact of *Echinacea* on cytokine changes in patients or models of COVID-19. Many of the human studies involved healthy participants or participants with relatively mild infections such as the common cold. The animal and cell culture studies used a variety of immune stimulating agents such as lipopolysaccharide (LPS), bacterial and viral infections. While animal models of cytokine storm exist [137], none were used by the studies included in the present review. These factors may decrease the generalizability of the findings to the treatment of COVID-19.

Similarly, the studies did not assess the changes in cytokine levels in models of cytokine storm. Cytokine storm is a complex syndrome involving cascades of interdependent inflammatory mediators which changes over the course of clinical progression. Defining this condition has been challenging due to the difficulty of differentiating a dysregulated immune response from a physiologic response to a severe infection [7]. Cytokines play an important role in the host response to an infection but at the same time, may cause harm to the host when released in excess. It has been hypothesized that inhibition of cytokine signaling could impair clearance of SARS-CoV-2, and result in worse outcomes such as secondary infections; this has been previously observed in the treatment of influenza [138] and subsequent to the use of IL-6 inhibitors in COVID-19 patients [133]. These findings may suggest that immune modulation may be appropriate for only a subgroup of COVID-19 patients. Additionally, cytokine production varies over the course of the response to the pathogen. Ideally, the immune response should be proportionate to the severity of the infection and result in a return to homeostasis following clearance of the pathogen [7]. The importance of timing may be relevant to interpreting the findings of the present review. The included studies measured cytokine levels at a variety of timepoints in the course of an infection; the impact of timing may account for some of the heterogeneity in the results presented. It has been hypothesized that the cytokine storm seen in COVID-19 occurs in two stages. The first stage is an underactive initial immune response which fails to adequately clear the virus. Subsequently, in response to the failed clearance, there is an overactive immune response [139]. Changes in the immune response at different time points in the course of disease progression suggest that the timing of different immunomodulatory therapies may be highly important [139].

## 5 Conclusion

The findings of the present systematic review suggest that the effect of *Echinacea* supplementation on cytokines may be predominantly anti-inflammatory, including the inhibition of cytokines that play a key role in the progression of severe COVID-19. Investigation of the potential therapeutic role of *Echinacea* supplementation in the prevention or treatment of cytokine storm due to COVID-19 may be warranted.

## Funding

No funding was received for the conduct of this research.

## Author contributions

The project was conceived by MA, KC and VC. MA, KC and VC developed the study protocol. The search strategy was conducted by VC. Data extraction was completed by all authors. Preliminary data analysis was completed by MA. All authors contributed to manuscript preparation and approved the final manuscript draft.

## Declaration of competing interest

The authors declare no conflict of interest.
