# Supplementary material for: A systematic review on the effects of Echinacea supplementation on cytokine levels: Is there a role in COVID-19?
Source: Metabol Open. 2021 Jul 29;11:100115. doi: 10.1016/j.metop.2021.100115 (PMC8320399; doi:10.1016/j.metop.2021.100115)
Supplement: Multimedia component 1 [file mmc1.docx]

Supplemental Materials

Supplemental File 1: List of excluded studies

| **Reference** | **Reason for exclusion** |
| --- | --- |
| Barrett B, Brown R, Voland R, Maberry R, Turner R. Relations among questionnaire and laboratory measures of rhinovirus infection. European Respiratory Journal. 2006 Aug 1;28(2):358-61. | Wrong outcomes |
| Wijesundara NM, Sekhon-Loodu S, Rupasinghe HV. Phytochemical-rich medicinal plant extracts suppress bacterial antigens-induced inflammation in human tonsil epithelial cells. PeerJ. 2017 Jun 22;5:e3469. | Wrong intervention |
| Pillai S, Pillai C, Mitscher LA, Cooper R. Use of quantitative flow cytometry to measure ex vivo immunostimulant activity of Echinacea: The case for polysaccharides. The Journal of Alternative and Complementary Medicine. 2007 Aug 1;13(6):625-34. | Wrong outcomes |
| Skopińska-Różewska E, Wasiutyński A, Sommer E, Skopiński P, Pastewka K, Zdanowski R, Bany J. Experimental immunology Modulatory effect of Echinacea pallida on cellular immunity and angiogenesis in mice. Central European Journal of Immunology. 2011;36(1):18-23. | Wrong outcomes |
| Lupfer C, Besnouin D, Tepper SE, Maselko M, Patton KM, Pastey M. Increased survivorship and altered cytokine profile from treatment of influenza A H1N1-infected mice with ekybion: a drug complex of natural extracts and inorganic compounds. Evidence-Based Complementary and Alternative Medicine. 2010;2011. | Wrong intervention |
| Schwarz E, Parlesak A, Henneicke-von Zepelin HH, Bode JC, Bode C. Effect of oral administration of freshly pressed juice of Echinacea purpurea on the number of various subpopulations of B-and T-lymphocytes in healthy volunteers: results of a double-blind, placebo-controlled cross-over study. Phytomedicine. 2005 Sep 15;12(9):625-31. | Wrong outcomes |
| Wang MJ, Raza SH, Wu Q, Xue CH, Liu JH, Zhang LF, Zhang WY, Wang AC, Wu H. Cichoric acid from extracted Echinacea purpurea induces the proliferation and apoptosis of peripheral blood mononuclear cells from yaks. Electronic Journal of Biotechnology. 2020 Sep 1;47:17-28. | Wrong outcomes |
| Morazzoni P, Cristoni A, Di Pierro F, Avanzini C, Ravarino D, Stornello S, Zucca M, Musso T. In vitro and in vivo immune stimulating effects of a new standardized Echinacea angustifolia root extract (Polinacea™). Fitoterapia. 2005 Jul 1;76(5):401-11 | Duplicate |
| Ritchie MR, Gertsch J, Klein P, Schoop R. Effects of Echinaforce® treatment on ex vivo-stimulated blood cells. Phytomedicine. 2011 Jul 15;18(10):826-31. | Duplicate |
| McCann DA, Solco A, Liu Y, Macaluso F, Murphy PA, Kohut ML, Senchina DS. Cytokine-and interferon-modulating properties of Echinacea spp. root tinctures stored at− 20° C for 2 years. Journal of Interferon & Cytokine Research. 2007 May 1;27(5):425-36. | Duplicate |
| Senchina DS, McCann DA, Asp JM, Johnson JA, Cunnick JE, Kaiser MS, Kohut ML. Changes in immunomodulatory properties of Echinacea spp. root infusions and tinctures stored at 4 C for four days. Clinica chimica acta. 2005 May 1;355(1-2):67-82. | Duplicate |
| Bodinet C, Lindequist U, Teuscher E, Freudenstein J. Effect of an orally applied herbal immunomodulator on cytokine induction and antibody response in normal and immunosuppressed mice. Phytomedicine. 2002 Jan 1;9(7):606-13. | Wrong intervention |
| Schapowal A. The triple action of the herbal medicine Echinaforce® in the treatment of colds and flu-like infections. Schweizerische Zeitschrift für Ganzheitsmedizin/Swiss Journal of Integrative Medicine. 2011;23(1):40-4. | Wrong study design |
| Elsässer‐Beile U, Willenbacher W, Bartsch HH, Gallati H, Mönting JS, Von Kleist S. Cytokine production in leukocyte cultures during therapy with Echinacea extract. Journal of Clinical Laboratory Analysis. 1996;10(6):441-5. | Wrong intervention |
| Yin SY, Wang WH, Wang PH, Aravindaram K, Hwang PI, Wu HM, Yang NS. Stimulatory effect of Echinacea purpurea extract on the trafficking activity of mouse dendritic cells: revealed by genomic and proteomic analyses. BMC genomics. 2010 Dec;11(1):1-8. | Wrong outcomes |
| Wijesundara NM, Sekhon-Loodu S, Rupasinghe HV. Phytochemical-rich medicinal plant extracts suppress bacterial antigens-induced inflammation in human tonsil epithelial cells. PeerJ. 2017 Jun 22;5:e3469 | Duplicate |
| Chicca A, Raduner S, Pellati F, Strompen T, Altmann KH, Schoop R, Gertsch J. Synergistic immunopharmacological effects of N-alkylamides in Echinacea purpurea herbal extracts. Planta Medica. 2009 Jul;75(09):PF12. | Duplicate |
| Bałan BJ, Sokolnicka I, SkopińSka-różEwSka E, Skopiński P. The modulatory influence of some Echinacea-based remedies on antibody production and cellular immunity in mice. Central-European journal of immunology. 2016;41(1):12. | Wrong outcomes |
| Mucci I, Legitimo A, Compagnino M, Pellati F, Nieri P, Benvenuti S, Metelli M, Longoni B, Mosca F, Consolini R. Root extracts of three species of Echinacea affect the maturation state of generated-monocyte human dendritic cells. | Conference/meeting abstract |
| Zhang X, Rizshsky L, Hauck C, Qu L, Widrlechner MP, Nikolau BJ, Murphy PA, Birt DF. Bauer Ketones 23 and 24 Contribute to Anti‐inflammatory Activity of Echinacea paradoxa paradoxa. | Conference/ meeting abstract |
| Fink C, Bonaterra GA, Kelber O, Weiser D, Kinscherf R. In vitro effect of STW11 on human dendritic cells. InNAUNYN-SCHMIEDEBERGS ARCHIVES OF PHARMACOLOGY 2012 Mar 1 (Vol. 385, No. SUPPL 1, pp. 26-26). ONE NEW YORK PLAZA, SUITE 4600, NEW YORK, NY, UNITED STATES: SPRINGER. | Conference/ meeting abstract |
| Soeberdt M, Oláh A, Knie U, Dähnhardt-Pfeiffer S, Bíró T, Abels C. 260 Anti-inflammatory and anti-pruritic properties of a lipophilic Echinacea purpurea root extract. Journal of Investigative Dermatology. 2016 May 1;136(5):S46. | Conference/ meeting abstract |
| Senchina DS, Wu L, Flinn GN, Konopka DN, McCoy JA, Widrelechner MP, Wurtele ES, Kohut ML. Year-and-a-half old, dried Echinacea roots retain cytokine-modulating capabilities in an in vitro human older adult model of influenza vaccination. Planta medica. 2006 Oct;72(13):1207. | Irrelevant erratum |
| Katryniok C, Bonaterra G, Weiser D, Kelber O, Kinscherf R. FOCUSED CONFERENCE GROUP: P09-NFLAMMATION AND IMMUNOPHARMACOLOGY: NEW TOOLS FOR OLD DISEASES INFLUENCE OF STW 11, A FIXED COMBINATION MEDICINAL PRODUCT, ON CYTOKINE PRODUCTION OF HUMAN MACROPHAGES: Paper No.: 2370. Basic & Clinical Pharmacology & Toxicology. 2010 Jul;107. | Conference/ meeting abstract |
| Cecil C, Oyegunwa A, Kandhi V, Eads D, Hansen R, Sikes M, Scholle F, Petty I, Davis J, Cech N, Laster S. Botanical medicines and their effects on the replication of influenza A virus and the influenza A virus-induced production of inflammatory mediators (92.14). | Conference/ meeting abstract |
| Lotts T, Ständer S, Soeberdt M, Nicolussi S, Guhl S, Maurer M, Abels C, Gertsch J. FAAH-and AMT-inhibitors exhibit anti-inflammatory and antipruritic effects: a role of the endocannabinoid system in atopic dermatitis: P246. Experimental Dermatology. 2012 Mar;21(3). | Conference/ meeting abstract |
| Bodinet C, Willigmann I, Beuscher N. Host-resistance increasing activity of root extracts from Echinacea species. Planta Medica. 1993 Dec;59(S 1):A672-3. | Conference/ meeting abstract |
| Bodinet C, Beuscher N. Antiviral and immunological activity of glycoproteins from Echinacea purpurea radix. Planta medica. 1991 Dec;57(S 2):A33-4 | Conference/ meeting abstract |
| Soeberdt M, Knie U, Metze D, Abels C. Anti-inflammatory and anti-pruritic activity of an alkylamide from Echinacea purpurea in murine models of delayed-type hypersensitivity and atopic dermatitis: P082. Experimental Dermatology. 2014 Mar;23(3). | Conference/ meeting abstract |
| Katryniok C, Bonaterra GA, Traut U, Hollender R, Weiser D, Kelber O, Kinscherf R. Influence on LPS-activated and not activated human macrophages by STW 11, a fixed combination medicinal product used in common cold and influenza. InNAUNYN-SCHMIEDEBERGS ARCHIVES OF PHARMACOLOGY 2010 Mar 1 (Vol. 381, pp. 41-41). 233 SPRING ST, NEW YORK, NY 10013 USA: SPRINGER. | Conference/ meeting abstract |
| Hushmendy S, Jayakumar L, Hahn A, Bhoiwala D, Bhoiwala D, Crawford D. The potential use of diet for rational gene targeting in treating genetically‐defined pathologies. The FASEB Journal. 2009 Apr;23:LB433-. | Conference/ meeting abstract |
| Soeberdt M, Olah A, Knie U, Metze D, Biro T, Abels C. Anti-inflammatory activity of alkylamides from Echinacea purpurea in keratinocytes in vitro and in mouse models of inflammatory skin diseases. InJOURNAL OF INVESTIGATIVE DERMATOLOGY 2014 May 1 (Vol. 134, pp. S14-S14). 75 VARICK ST, 9TH FLR, NEW YORK, NY 10013-1917 USA: NATURE PUBLISHING GROUP. | Conference/ meeting abstract |
| Yang NS, Wang CY, Chiu SC, Shyur LF. Functional genomics of immuno-modulatory activities of medicinal plant extracts/phytocompounds in human dendritic cells/monocytes. Planta Medica. 2009 Jul;75(09):SL45. | Conference/ meeting abstract |
| Barak V, Birkenfeld S, Halperin T, Kalickman I. The effect of herbal remedies on the production of human inflammatory and anti-inflammatory cytokines. The Israel Medical Association journal: IMAJ. 2002 Nov 1;4(11 Suppl):919-22. | Wrong intervention |
| Senchina DS, Wu L, Flinn GN, Konopka DN, McCoy JA, Widrelechner MP, Wurtele ES, Kohut ML. Year-and-a-half old, dried Echinacea roots retain cytokine-modulating capabilities in an in vitro human older adult model of influenza vaccination. Planta medica. 2006 Oct;72(13):1207. | Duplicate |
| Barrett B, Brown R, Rakel D, Rabago D, Marchand L, Scheder J, Mundt M, Thomas G, Barlow S. Placebo effects and the common cold: a randomized controlled trial. The Annals of Family Medicine. 2011 Jul 1;9(4):312-22. | Wrong outcomes |
| de Oliveira CC, Abud AP, de Oliveira SM, de SF Guimarães F, de Andrade LF, Di Bernardi RP, Ediely LD, Kuczera D, Da Lozzo EJ, Gonçalves JP, da S Trindade E. Developments on drug discovery and on new therapeutics: highly diluted tinctures act as biological response modifiers. BMC complementary and alternative medicine. 2011 Dec;11(1):1-1. | Wrong intervention |
| Sharma M, Arnason JT, Hudson JB. Echinacea extracts modulate the production of multiple transcription factors in uninfected cells and rhinovirus‐infected cells. Phytotherapy Research: An International Journal Devoted to Pharmacological and Toxicological Evaluation of Natural Product Derivatives. 2006 Dec;20(12):1074-9. | Wrong outcomes |
| Jia QH, Li QL, Wu TL, Zhang ZQ, Shi QM, Liu ZJ. Anti-Inflammatory Effect of Echinacea purpurea Polysaccharide (EPS) on the Pulmonary Macrophage cell line Raw264. 7 in Mice. LATIN AMERICAN JOURNAL OF PHARMACY. 2018 Jan 1;37(4):733-9. | Unable to get full text or response from author |
| Beuscher N, Bodinet C, Willigmann I, Egert D. Immune modulating properties of root extracts of different Echinacea species. Zeitschrift Phytotherapie. 1995;16:157-66. | Unable to get full text or response from author |
| Shi Q, Lang W, Wang S, Li G, Bai X, Yan X, Zhang H. Echinacea polysaccharide attenuates lipopolysaccharide‑induced acute kidney injury via inhibiting inflammation, oxidative stress and the MAPK signaling pathway. International Journal of Molecular Medicine. 2021 Jan 1;47(1):243-55. | Duplicate |
| Gerstel J, Langland J. Echinacea purpurea: Deciphering the Controversy Behind Its Medicinal Properties. Sage Journals. 2018 Sept 21. | Conference/ meeting abstract |

### Supplemental File 2: Risk of Bias Assessment for Pre/Post and Animal Studies

 NIH Quality Assessment for pre-post human studies with no control group

| **Study** | **1. Study Question** | **2. Eligibility Criteria** | **3. Representativeness** | **4. All eligible participants enrolled** | **5. Sample Size** | **6. Intervention description** | **7. Outcome** | **8. Blinding** | **9. Follow up** | **10. Statistic** | **11. Multiple baselines** | **12. Individual data** | **Overall** |
| --- | --- | --- | --- | --- | --- | --- | --- | --- | --- | --- | --- | --- | --- |
| Dall’Acqua, 2015 | Yes | Yes | CD | NR | No | Yes | Yes | No | NR | Yes | Yes | NA | Fair |
| Guiotto, 2008 | Yes | No | CD | NR | No | Yes | Yes | No | NR | Yes | Yes | NA | Fair |
| Randolph, 2003 | Yes | Yes | Yes | NR | No | Yes | Yes | No | NR | Yes | Yes | NA | Poor |

NR: not reported

OHAT risk of bias assessment for animal studies

| **Author, Year** | **Selection Bias** | | **Performance Bias** | | **Attrition/ Exclusion Bias** | **Detection Bias** | | | **Other** |
| --- | --- | --- | --- | --- | --- | --- | --- | --- | --- |
|  | 1. Adequately randomization | 2. Allocation to study group concealment | 5. Identical experimental conditions across study groups | 6. Research personnel blinding | 7. Complete outcome data | 8. Confident in the exposure characterization | 9. Confident in the outcome assessment | 10. all Measured outcomes reported | 11. Other potential threats to internal validity |
| Abdelmonem, 2015 | Def low | Prob high | Def low | High-NR | Prob low | Def low | Def low | Def low | Def low |
| Abdallah, 2015 | Prob low | High-NR | Def low | High-NR | High-NR | Prob low | Prob low | Prob low | Prob low |
| Abdel Rahman, 2018 | Prob low | High-NR | Def low | High-NR | Prob low | Def low | Def low | Def low | Def low |
| Cundell 2003 | Def low | Def low | Prob high | Def low | Prob low | Def low | Def low | Def low | Def low |
| Dogan, 2014 | Def low | Prob high | Def low | High-NR | Def low | Prob low | Def low | Def low | Prob low |
| Fusco 2010 | Def high | Prob high | Prob low | High-NR | High-NR | Prob low | Prob low | Prob low | Prob high |
| Ghaemi, 2009 | Prob high | Prob high | Prob low | High-NR | Def low | Def low | Def low | Def low | Prob low |
| Goel, 2002 | Prob low | Def low | Def low | High-NR | Def low | Def low | Def low | Def low | Def low |
| Goel 2002 (#147) | Prob low | High-NR | Def low | High-NR | Prob low | Def low | Def low | Prob low | Def low |
| Hayashi, 2001 | Prob high | Prob high | Def low | High-NR | Def low | Def low | Def low | Def low | Prob high |
| Jiang 2014 | Def low | Def low | Def low | High-NR | Prob low | Def low | Def low | Def low | Def low |
| Liu, 2012 | Prob low | Prob low | Def low | High-NR | Prob low | Prob low | Def low | Def low | Def low |
| Liu, 2017 | Prob high | Prob high | Def high | High-NR | Prob low | Def low | Def low | Def low | Prob high |
| Li, 2020 | Prob low | High-NR | Prob low | High-NR | Prob low | Prob low | Prob low | Prob low | Prob low |
| Park, 2018 | Prob low | High-NR | Def low | High-NR | Prob low | Def low | Def low | Def low | Def low |
| Sgorlon, 2016 | Prob low | High-NR | Probably high | High-NR | Prob low | Prob low | Prob low | Def low | Prob high |
| Shi, 2020 | Prob low | High-NR | Prob low | High-NR | Prob low | Prob low | Prob low | Prob low | Prob low |
| Sutovska, 2015 | High-NR | High-NR | High-NR | High-NR | High-NR | Prob low | Prob low | Def low | Prob high |
| Turkistani, 2019 | High-NR | High-NR | Prob low | High-NR | Prob low | Prob low | Prob low | Prob low | Prob low |
| Uluisik, 2012 | High-NR | High-NR | Prob low | High-NR | Prob high | Prob low | Prob low | Def low | Prob high |
| Yamada, 2011 | Prob high | Prob high | Prob low | High-NR | Prob low | Def high | Def low | Def low | Def low |
| Yu, 2013 | Prob low | High-NR | Probably low | High-NR | High-NR | Prob low | Def low | Def low | Prob low |
| Zhai, 2007 | Prob low | High-NR | Probably high | High-NR | High-NR | Def low | Def low | Def low | Prob low |
| Zhang, 2020 | Prob low | High-NR | Prob low | High-NR | Prob low | Prob low | Prob low | Prob low | Prob low |

Prob: probably, Def: definitely, NR: not reported
